# Multi-Analytical and Non-Invasive Approach for Characterising Blackened Areas of Originally Blue Paints

**DOI:** 10.3390/molecules29246043

**Published:** 2024-12-22

**Authors:** Maria Labate, Maurizio Aceto, Giacomo Chiari, Simone Baiocco, Lorenza Operti, Angelo Agostino

**Affiliations:** 1 Dipartimento di Chimica, Università degli Studi di Torino, Via P.Giuria 7, 10125 Torino, Italy; maria.labate@unito.it (M.L.); lorenza.operti@unito.it (L.O.); 2 Dipartimento per lo Sviluppo Sostenibile e la Transizione Ecologica (DISSTE), Università degli Studi del Piemonte Orientale “Amedeo Avogadro”, Piazza S. Eusebio 5, 13100 Vercelli, Italy; 3 Getty Conservation Institute (Retired), 1200 Getty Center Drive, Los Angeles, CA 90049, USA; gc.giacomochiari@gmail.com; 4 Palazzo Madama—Museo Civico d’Arte Antica, Piazza Castello, 10122 Torino, Italy; simone.baiocco@fondazionetorinomusei.it

**Keywords:** azurite, blue paintings, non-invasive analysis, FORS, XRF, XRD, elemental mapping

## Abstract

Azurite, a natural mineral pigment consisting of basic copper carbonate (2CuCO_3_·Cu(OH)_2_), is one of the Middle Ages’ most common blue pigments. Why paintings originally coated with azurite appear blackened today remains debated. Using a non-invasive multi-analytical approach, the study analysed several unexpectedly black-appearing details (objects such as books or clothing such as veils, robes, or mantles) in Antoine de Lonhy’s works. The aim was to investigate if the black colour was due to intentional iconographic reasons, incautious restoration work, or painting deterioration. The analytical results displayed the presence of the blue pigment azurite, therefore, the expected original colour of various areas should be blue. To shed light on the discussion regarding the blackening, several other Renaissance paintings with similar black details were analysed, all from the same period and geographic area as de Lonhy’s works and conserved under identical conditions. The reasons why the blackening takes place are still unclear. However, the combined use of X-ray fluorescence spectrometry (XRF), UV-visible diffuse reflectance spectrophotometry with optical fibres (FORS), portable X-ray diffraction (XRD), and the elemental mapping based on the XRF data revealed that these blackened areas were originally painted with azurite, suggesting they were once blue. This finding significantly changes the overall appreciation of these artworks.

## 1. Introduction

A multidisciplinary study of artworks attributed to Antoine de Lonhy (ca. 1446–1490) [1] was facilitated by a 2021 exhibition dedicated to the artist, held in Palazzo Madama (Museo Civico d’Arte Antica di Torino). Antoine de Lonhy was an eclectic travelling painter previously known as Master of the Trinity of Turin. Trained in the extraordinary environment of the Flemish and French artists and culture in the middle of the 15th century in Bourgogne, the artist worked then in Toulouse and Barcelona, ending his career in the Duchy of Savoy, where he left a significant legacy. Different types of his artwork, such as manuscripts, frescoes, and panel paintings, were analysed through non-invasive techniques to define the artist’s colour palette. Except for the illuminated manuscripts, in which the blue plays a role of particular relevance [2], the absence of this colour is immediately noticeable in all other artworks considered. In most cases, clothes, mantles, and the Virgin’s veils appear unusually coloured in black. Other coeval paintings preserved at Palazzo Madama, for which darkening occurred, were also considered.

According to the literature, a large number of black areas in mediaeval paintings were originally blue [3] and made of azurite (2CuCO_3_·Cu(OH)_2_), the most widespread mediaeval blue pigment. This is a valuable mineral from different localities of Europe [4,5,6,7,8] often used together with the more expensive ultramarine blue, obtained from Lapis Lazuli. As is well known, azurite is a low covering pigment, so it needs several coats of it and is always laid onto black (*veneda*, consisting of carbon black or vine black), red-brown (*morellone*, made of earths and ochres with carbon black) layers in the frescos, and different colour backgrounds were also used in panel paintings [9]. Pictorial layers with azurite are not the only ones to show blackening since similar behaviour has also been studied for Egyptian blue [10].

The blackened appearance of azurite paintings can be attributed to endogenous and exogenous causes, or other factors, i.e., due to the presence of other materials associated with it, such as binders or varnishes [3,11,12,13,14,15,16,17,18,19,20,21], to dust accumulation [22], or to underlying preparatory layer becoming visible because of the porosity of the azurite layers [3]. The darkening of azurite itself may depend on its preparation process and particle size [20,23,24,25,26,27] or on its conversion into other minerals due to interaction with the environment. The main black alteration products are tenorite [28,29,30], covellite [31], or copper carbamates [9]. The alteration can be induced by exposure to hydrogen sulphide gas and moisture [31,32,33] by heating treatment such as fires [34,35,36,37,38,39], or by the use of alkaline pH cleaning materials [12,39,40]. Many possible concurrent issues are discussed in the literature [41]. Still, no definitive answer is given about the darkening because of the uncertainty on the pigment purity, the binder employed, or the artistic technique adopted [42,43], the presence of a preparation layer and/or a varnish, and the artwork conservation context.

The paper involves ten paintings attributed to Antoine de Lonhy and twenty works painted by coeval artists. This study aims to characterise the blackened areas of various 15th- and 16th-century paintings using a totally non-invasive approach. The techniques adopted allow very fast, cost-effective, and easy measurements to be carried out in situ. Furthermore, possible identification of pigments in the blackened painted areas may provide important information for defining the original palette of the artworks, their conservation history, and useful indications for future restoration work (e.g., for defining pictorial integrations or for adopting the most suitable products in cleaning). To characterise possible blackening due to the alkalinity of restoration products, the behaviour of azurite in alkaline environments was tested by experiments conducted on azurite mock-ups using a restoration product of common historical practice.

## 2. Results

**Figure 1:**
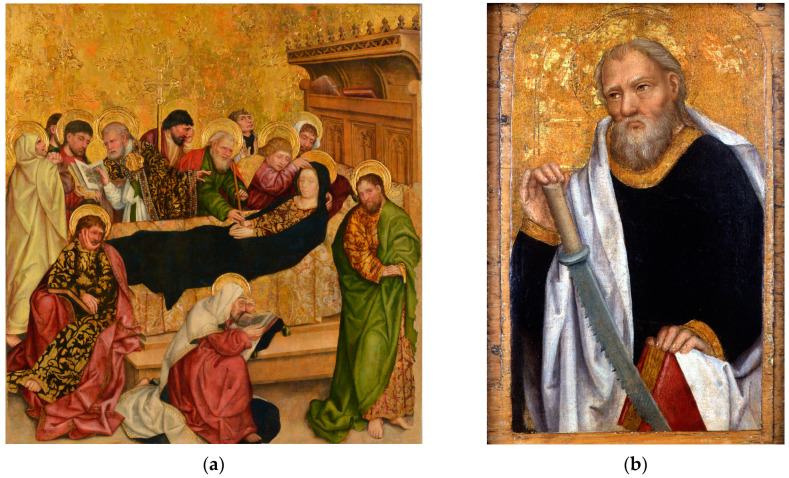
The Virgin’s mantle in Death of the Virgin (**a**) and Simon’s tunic (inv. 5/C) in Apostles Predella (**b**) are good examples showing the recurring dark and rather flat coats under study.

In the illuminated manuscripts attributed to Antoine de Lonhy (inv. 399), the blue colour plays a predominant role and, in contrast, it seems to be almost absent from several other artworks of the same epoch exhibited at Palazzo Madama. The historical blue pigments found in the in Book of Hours’ palette are azurite and ultramarine [2]. The naked-eye impression of details of most of other works such as ropes, veils, mantles, and some objects like books is perplexing since they appear almost black. The surface looks flat, without the volume rendering of drapery which can instead be appreciated in the remaining non-blue parts of the paintings (Figure 1).

**Figure 2:**
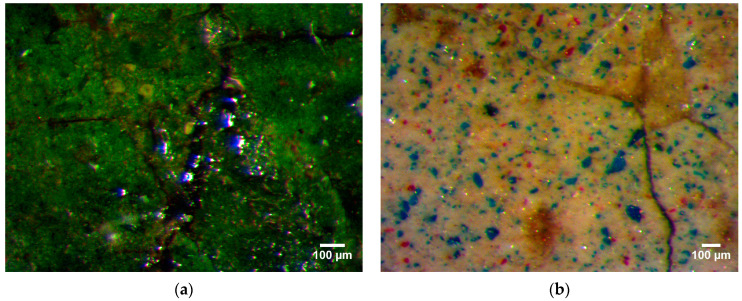
Images acquired by stereo microscopy on painting details of apostle Matthew’s robe (**a**) (inv. 5/A) and apostle Simon’s mantle (**b**) (inv. 5/C). In these areas, blue pigment is present to improve the colour in the greens and whites.

**Figure 3:**
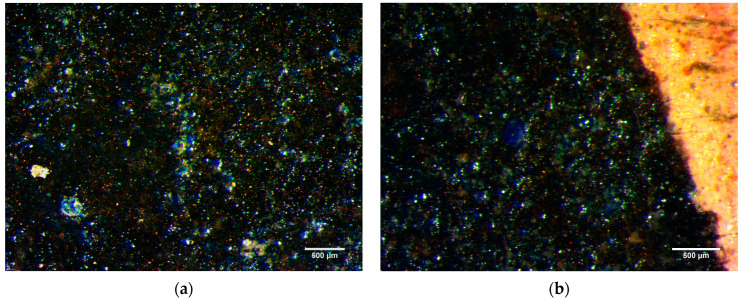
Apostle Peter’s mantle details (inv. 5/B) by stereo microscopy. The open texture of the blue coat in which pigment particles have fine (a)-to-coarse (b) size.

Stereomicroscope image acquisition was only possible on paintings of the Apostles predella (inv. 5) because of steric and handling reasons. Grains of a blue pigment were detected in green, white, and grey areas (Figure 2). Many blue pigment particles can also be found in dark parts. Some variability in pigment size is observed as well (Figure 3).

Several points in dark areas were analysed by XRF and, in most cases, copper was detected as shown in Table 1. In the 37 areas identified in 26 paintings, the copper was detected in 32 cases and in 5 cases it was not detected or detected in a concentration lower than 1%. These are God’s mantle of 470/D, Madonna’s veil of 428/D, the crow of 448/D, the robe of the bottom left figure of 460/D, and Saint Monica’s veil of 787/D.

**Figure 4:**
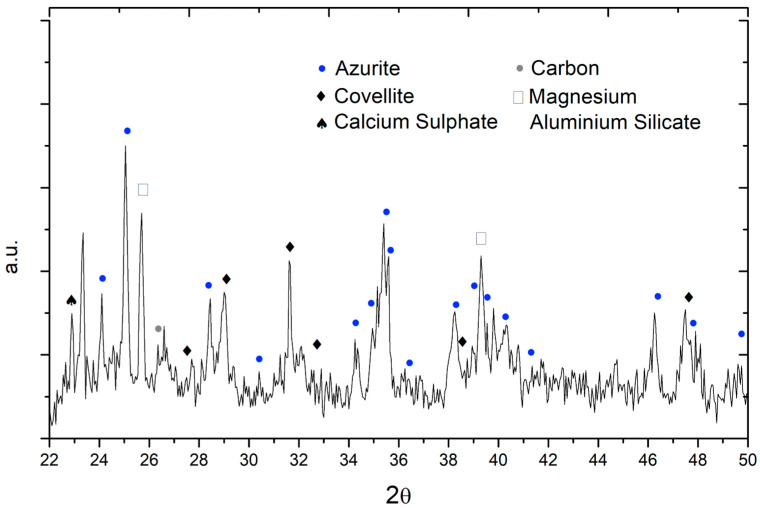
Diffraction pattern obtained from portable XRD analysis (Duetto) of Simon’s tunic (inv. 5/C). Dominant azurite (PDF number: 00-011-0682) with covellite (PDF number: 00-006-0464), carbon (PDF number: 00-026-1076), calcium sulphate (PDF number: 00-026-0328) and magnesium aluminium silicate (PDF number: 00-027-0716).

XRD analysis was performed only on the Apostles’ panels. The main phase recognisable in the diffraction pattern obtained on Simon’s tunic (inv. 5/C) is azurite with a minor amount of covellite, carbon, calcium sulphate, and magnesium aluminium silicate mineral phases (Figure 4).

**Figure 5:**
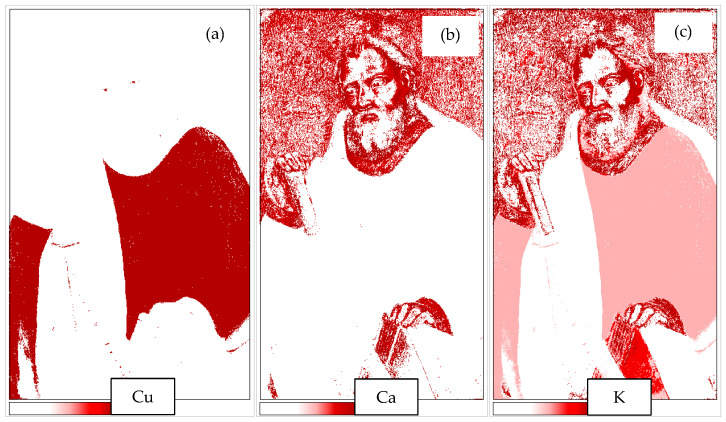
SmARTscan map of St. Simon (inv. 5/C) for copper (**a**), calcium (**b**), and potassium (**c**).

For steric reasons, the number of measurements for Duetto’s XRD was not sufficient to generate maps of compounds using SmARTscan. The XRF measurements were on the contrary sufficient. We report here the maps of copper for Apostle Simon since this painting has the most extended regions of darkened blue (Figure 5a).

**Table 1:** FORS and XRF results of the measurement areas of paintings in which unintentional or probably deliberate darkening was observed. The ‘unknown’ reflectance spectrum type refers to that with the spectrum characteristics in Figure 6, with an increase in reflectance in the near-infrared (NIR) from about 850–900 nm.

Catalogue Code	Detail	FORS Results		XRF Results
		Num.	Attribution	Detection of Copper
Inv. 5/A	book	1	Carbon black	NA *
		2	Unknown	X
Inv. 5/B	Mantle	1	Unknown	X
Inv. 5/C	Tunic	1	Unknown	X
Inv. 5/D	Mantle	1	Unknown	X
470/D	God’s mantle	1	Carbon black	
		2	Unknown	X
BBC	Madonna’s veil	1	Unknown	X
	Mantle bottom center	2	Unknown	X
	Pillow	3	Unknown	X
418/D	Madonna’s veil	1	Unknown	X
419/D	St. Anna’s mantle	1	Unknown	X
	Madonna’s mantle	2	Unknown	X
428/D	Madonna’s veil	1	Carbon black	
431/D	Madonna’s veil	1	Azurite	X
433/D	Madonna’s veil	1	Unknown	X
434/D	Madonna’s veil	1	Unknown	X
436/D	Jesus’ tunic	1	Azurite	X
	Central fig.’s mantle	2	Unknown	X
441/D	S. Caterina sleeves	1	Azurite	X
442/D	Madonna’s veil	1	Azurite, Unknown	X
448/D	Madonna’s veil	1	Azurite	X
	Crow	2	Carbon black	
460/D	Bottom left fig.	1	Carbon black	
462/D	Madonna’s veil	1	Azurite	X
476/D	Madonna’s veil	1	Azurite	X
483/D	Madonna’s veil	1	Azurite	X
	Madonna’s veil (inner)	2	Unknown	X
489/D	Madonna’s veil	1	Unknown	X
491/D	Madonna’s veil	1	Unknown	X
512/D	Madonna’s veil	1	NA *	X
516/D	Jesus’ mantle	1	Azurite	X
	Maddalena’s dress	2	Unknown	X
721/D	Madonna’s veil	1	Azurite	X
		2	Unknown	X
774/D	Madonna’s veil	1	Unknown	X
	Mantle bottom left	2	Unknown	X
787/D	S. Monica’s veil	1	Carbon black	

* NA means not acquired.

Simon’s rope is visibly darkened, almost black. The elemental map showing the distribution of copper perfectly fits the tunic, indicating that copper is equally distributed over the whole region. It may be interesting to notice that the pupils and the sword also contain minor quantities of copper, while they are grey in the visible image. The fact that copper is ubiquitous and homogeneously distributed on the dark part may be a further indication that the darkening and azurite are connected. This is confirmed by the XRD measurement that shows, in addition to the dominant azurite, the presence of covellite and carbon.

**Figure 6:**
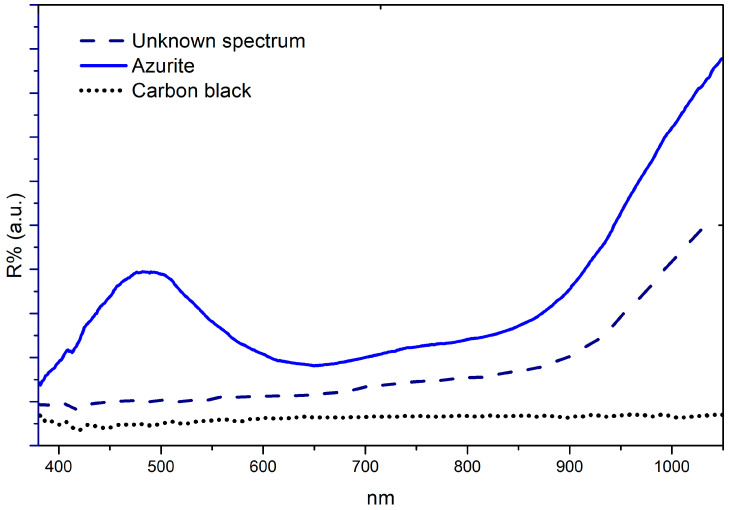
FORS spectra. Comparison among azurite, carbon black, and “unknown” reflectance spectra, acquired in correspondence with Simon’s tunic (inv. 5/C).

Calcium, shown in Figure 5b, may be in the form of calcite, as a pigment or as part of the priming. The map for potassium is also interesting (Figure 5c). The larger concentration of K is in the dark red parts, which is compatible with the hypothesis that it is part of the mordant of red lacquer. Considering the results of FORS analysis (Table 1), three types of reflectance spectra have been identified in dark areas, as shown in Figure 6:

- The first type of reflectance spectrum is characterised by low reflectance throughout the spectral range investigated. XRF’s non-detection of characteristic elements of other black pigments suggests it may be carbon black.
- Another rather recurrent type of spectrum is observed, consisting of a steady absorbance throughout the visible range (Vis) with an increase in reflectance in the near-infrared (NIR) from about 850–900 nm. These kinds of spectra are defined in Table 1 as “unknown”. XRF detected copper with well-defined signals in all corresponding areas while it was absent, or in irrelevant concentrations, in areas defined as carbon black.
- Despite the dark aspect, azurite is identified in some spectra by the typical reflectance minimum located at 640 nm [44]. This occurred in different measurements of Madonna’s veils (431/D, 442/D, 448/D, 462/D, 476/D, 483/D, and 721/D), Jesus’ tunic and mantle (436/D and 516/D) and Saint Caterina’s sleeve (441/D).

However, in 442/D, 483/D, and 721/D the spectrum defined as “unknown”, and the azurite spectrum, were both detected in different parts of the veils. In contrast, for two cases, both the reflectance spectrum attributable to carbon black and the “unknown” spectrum were detected (book of inv. 5A and God’s mantle of 470/D).

**Figure 7:**
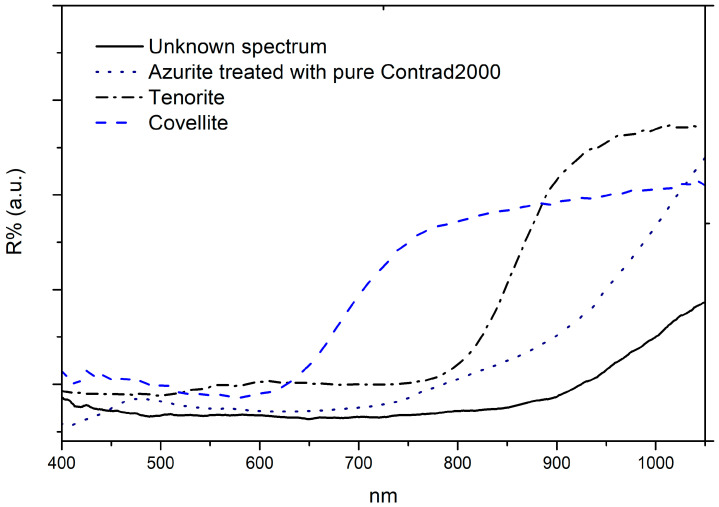
Comparison among tenorite (CuO), covellite (CuS), and azurite treated with pure Contrad 2000 and blackened areas reflectance spectrum, acquired in correspondence with Simon’s tunic (inv. 5/C).

The FORS spectra of the darkened areas were compared with the one measured on a tenorite (CuO) and covellite (CuS) samples. Furthermore, a comparison was made with reflectance spectra from azurite paintings treated with pure Contrad 2000 (Figure 7), the very alkaline restoration product described in Section 4.6.

## 3. Discussion

The study of Antoine de Lonhy’s colour palette applied to illuminated manuscripts (inv. 399) showed the presence of perfectly preserved azurite and ultramarine pigments, while a completely different *scenario* has been observed in the other panel paintings considered, by de Lonhy and other contemporaries.

Observing under the microscope the saint Apostles (inv. 5) a blue pigment is detected that was possibly intended for many purposes: to darken the green, to give a metallic look to some objects, such as Peter’s key (inv. 5/B) or Simon’s saw (inv. 5/C), and to give a colder tone to the white. The irregular shape and the heterogeneity of pigment particle size, appreciable under the microscope, points to its natural mineral origin, indicating that a restoration integration with synthetic fine material did not take place. The blue pigment particles observed in the dark backgrounds and traced back to azurite by XRD analysis do not refer to a coherent pictorial layering but rather to a residue that reveals underlying layers, suggesting that the original surface layers may have been removed.

Regarding dark areas of all the paintings considered and according to the results obtained from all the techniques adopted, in some cases, deliberate use of carbon black can be interpreted, but in other instances, the black, more likely, belonged to preparatory layers originally underlying the blue. Some objects and some robes such as the crow (448/D), the sky in a night scene (512/D) and the monastic robes (460/D and 787/D) are likely to be intentionally black. In the case of the Virgin’s veil (428/D), the visible black refers to a layer made to accommodate overlying paintings that have been lost. In the cases of the Madonna’s veil of the Death of the Virgin (BBC) and the God’s veil of Trinity (470/D), the black portions are undoubtedly pictorial integrations due to the restoration work where, again, the blue layers originally in azurite had a conservation history that has compromised them, leaving only a residue detected with XRF and FORS analysis. In any case, the colour of the restoration intervention was certainly tuned to the colour of the surrounding original, suggesting that this was already darker than blue.

Although no longer visible as blue, in many cases, reflectance spectra ascribable to azurite were obtained, however, most measurement points revealed a spectrum not traceable to any pigment according to the reference spectra database present in the literature. The reflectance spectra were considered as “unknown” perhaps due to the incompleteness of the available databases but also because of the difficulty in characterising very dark pigments or mixtures by reflectance analysis. In the case of this study, all the reflectance spectra defined “unknown” have the same characteristics in the NIR spectral range, as defined in the results section. The optical microscope observation of blue pigments, the systematic detection of copper by XRF and, in cases where the use of XRD was possible also the identification of mineral phases, suggest that every darkened area in which “unknown” reflectance spectra were detected contained azurite. Achieving this through a totally noninvasive approach is of particular importance because it allows quick and relatively easy identification of azurite where it is not, or no longer, easily detectable. This was made possible through the use of an extended set of paintings traceable to a similar period of production but with different conservation histories.

The possibility that other copper-based pigments or alterations of azurite may have played a significant role was considered. A possible candidate is Kassel brown, a mixture of black and red copper oxides [45]. This pigment, however, has spectral characteristics not found in our observations [46].

The comparison among the reflectance spectra of our tenorite sample, tenorite sample, the azurite painting treated with pure Contrad C2000, and the blackened azurite from the original work (Figure 7) show that the spectral features of tenorite are quite different. The same can be said from a comparison with the covellite reflectance spectrum, available also in the literature [47,48]. Tenorite was compared although it is not detected with XRD because it may be present below the detection limit. Even if tenorite and covellite were present in low concentration, they do not mainly contribute to characterise the reflectance spectrum as observed in the areas investigated with both XRD and FORS (Simon’s robe inv. 5/D) commented previously. On the other hand, the spectra obtained by applying Contrad 2000 on the azurite painting result in lowering the reflectance in the visible range, but not in the near-infrared range in a way comparable to the one shown in the blackened areas spectra.

**Figure 8:**
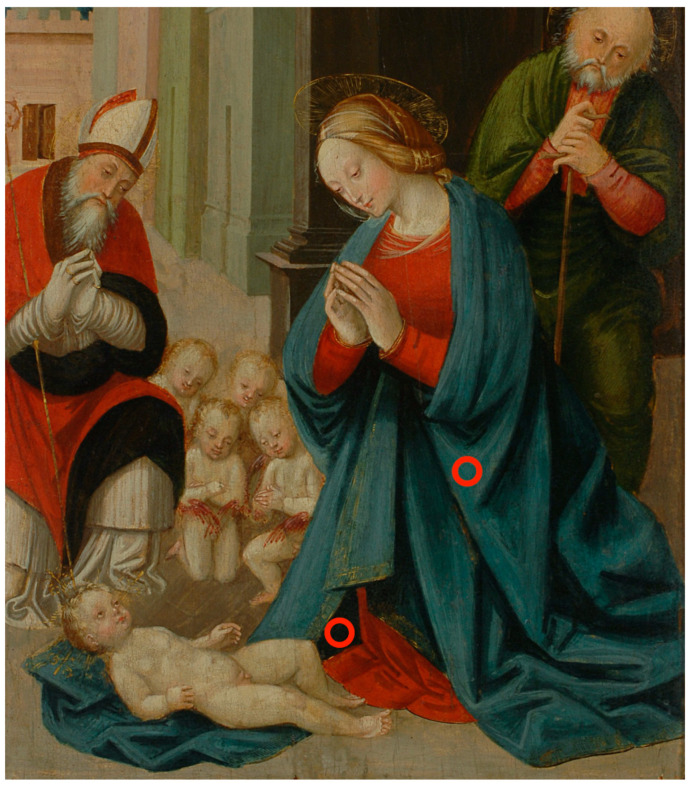
Measurement points (red circles) on the Virgin’s mantle in 483/D. FORS spectra acquired onto the light blue rope can be assigned to azurite while the dark inner part of the veil gives reflectance spectra of blackened azurite.

The causes of the darkening remain somehow elusive, but the detection of azurite and darkened areas with azurite in the same region as it happens in 483/D (Figure 8) and 721/D, might suggest that the darkening is not always due to an altered painting but more likely to the purpose of making some specific areas darker.

The darkened areas with azurite reflectance spectra could therefore be a result of the joint contribution of mixed carbon black and azurite to make some areas darker, while in some other cases the azurite layer has an open texture, due to the accidental partial loss of the pictorial layer, showing that the dark coating is an underlying painting layer laid as a background.

## 4. Materials and Methods

To define the materials of the darkened layers and to identify possible clues referable to any of the above-mentioned causes of the alteration process, a series of data were collected on dark areas of 30 paintings. A non-invasive and multi-analytical procedure was applied using portable instrumentation on the artworks listed in Table 2. Images of the works considered are available in Supplementary Materials. This type of approach was the only one possible since sampling was not permitted. Instrumental specifications are listed below.

FORS spectrophotometry and X-ray fluorescence spectrometry instruments have been described in detail in Agostino et al. [2]. The depth of information obtained by these analyses is different: in the first case, the surface is investigated (in a wavelength-dependent manner), and in the second case varying thicknesses could be analysed, depending on the excitation energy, the composition, and the density of the pictorial layers. According to various experiments, the thickness of hundreds of micrometres can be measured to detect information by reaching the preparatory layer of the painting. The portable XRD/XRF instrument DUETTO is well described by Chiari et al. [49]. The adopted analytical techniques do not allow for obtaining information about the binders, even though the investigated historical period includes the transition from the use of *tempera* to *tempera grassa* and oil techniques. There are no exhaustive reports of previous analyses of these artworks in the literature. Moreover, the documentation of the painting’s conservation history is known and detailed in a few cases, but in most situations, previous restorations are poorly documented. For example, *Trinity* (Palazzo Madama no. 470/D) and *Death of the Virgins* (private collection) were both transferred from panel to canvas, for conservation reasons, without any description.

### 4.1. Optical Microscopy (OM)

For OM a Leica MZ16 stereo microscope (Wetzlar, Germany) with 45° illumination was used, with Light source Leica Schott KL 1500 LCD. Microscope images were obtained exclusively from the Apostles panels (Palazzo Madama no. inv. 5/D) since their size permits the positioning of the areas of interest under the instrument.

### 4.2. X-Ray Fluorescence Spectrometry (XRF)

The elemental analyses were carried out by XRF. The instrumentation was a Thermo NITON spectrometer XL3T-900 GOLDD model (East Greenbush, NY, USA), equipped with an Ag anode, max. voltage 50 kV, max. current 100 µA and power 2 W, spot size 3 mm. The obtained spectra were processed with the commercial software BAxil v. 1.8 (Brightspec NV/SA, Belgium) derived from the academic software QXAS from IAEA.

### 4.3. UV-Visible-NIR Diffuse Reflectance Spectrophotometry with Optical Fibres (FORS)

FORS analysis was carried out using an Avantes AvaSpec-ULS2048XL-USB2 model spectrophotometer (Apeldoorn, The Netherlands) and AvaLight-HAL-S-IND tungsten halogen light source. The spectral range acquired is 375–1100 nm, spot size of 1.5 mm.

### 4.4. X-Ray Diffraction Analysis (XRD)

XRD analyses were performed using DUETTO, a portable instrumentation jointly developed by Examinart inXitu Inc. (Palo Alto, CA), and the Getty Conservation Institute (Los Angeles) designed specifically for non-invasive analysis of Cultural Heritage artefacts [44]. It consists of an X-ray source with copper target, voltage 30 kV, power 10 W and CCD detector. The analysis range is between 20° and 50° 2θ with step 0.05°, 2θ resolution from 0.15° to 0.5°. The ICDD (International Centre for Diffraction Data) diffractometric database JC-PDF2 was adopted for interpretation.

### 4.5. Elemental Maps Using the SmARTscan Procedure Based on XRF Measurements

The SmARTscan program [50] simulates the results of the macro-XRF scanners using a limited number of XRF measurements that are integrated with their position and colour. For each known point, six variables are available: X, Y, R, G, B, and the elemental concentration. All other unknown points miss the XRF results needed to generate the elemental map. This value is estimated from the shorter Euclidean distance from all the measured points in the 6-fold statistical space.

### 4.6. Mock-Up Samples for Comparison with the Originals and Conducting Cleaning Experimental Tests

For the Tenorite mock-up, copper(II) powder (Alfa Aesar, 44356) was used. The Covellite sample is a mineralogical specimen belonging to the collection of Museo Regionale di Scienze Naturali of Turin (code M/6913). Some painted samples with azurite (Azurite natural, standard, 0–120 µm, Kremer 10200) were made in egg yolk tempera (*Tempera grassa*). The coatings on the canvas or canvas board were treated with Contrad 2000 applied in varying dilutions (pure, 5% to 30%). To enhance the results, the product was tested in purity, a concentration theoretically not appropriate and not applied. For the same reasons in all tests, the product was applied but not rinsed off as would be required in its application. The making of the mock-ups was intentionally very simplified to test the interaction of azurite with a very basic material. For this reason, neither preparation nor paint was used, precisely to minimize the number of variables that play a role in any change in the pigment.

Contrad 2000 was a rather popular restoration product having an alkaline pH. Contrad consists of a complex emulsion in an aqueous base of anionic and non-ionic surfactants, stabilising agents, alkalis, detergents, and non-phosphate-based sequestering agents. Considering that pH is mentioned in the literature as one of the causes of blackening, Contrad 2000 was chosen because widely used in painting conservation in the past, to evaluate the effect on azurite of a product with an alkaline pH (pH > 13). Moreover, the paintings considered in this study may have been restored one or more times with alkaline products throughout their often poorly documented conservation history.

## 5. Conclusions

The present work defines a fast method for the detection of the pigment azurite even in those areas where the blue colour is no longer appreciable. Darkened areas on paintings were analysed by totally non-invasive techniques able to distinguish areas including an originally black pigment, from those presumed to be originally blue.

In some artworks, the observation of blue pigment grains by stereo microscope and the detection of copper by XRF point to the use of azurite, also confirmed by XRD, and azurite was shown to be the most abundant phase present. The FORS spectra show features comparable with very dark blue spectra and thanks to this study, they could refer to the painting layer in which azurite is present but no longer perceived as blue and as it was originally intended.

Since the works investigated have different conservation histories, various conditions may influence reflectance behaviour. The dark appearance of the painted areas could be attributable to several causes not inferable from the analyses adopted in this study: a surplus of darkened binder, the effect of the varnish, the possible presence of dark alteration products, and residual azurite with an open texture making the dark layer underneath visible or incautious cleaning. All this may be due, sometimes to conservative reasons, and at other times to deliberate artistic choices to achieve a darker rendering.

It is also possible to conclude that Antoine de Lonhy’s artworks had to be much bluer than what we observe today as it happens for many other works taken into consideration. The pictorial surfaces now visible appear to observers as black and with no volume, but must originally have had pictorial layers that have now been lost.

## Figures and Tables

**Table 2:** List of the artworks analysed in this study with the code of the Museo Civico d’Arte Antica catalogue available online.

Title/Date	Type	Author	Catalogue Code
Sei apostoli/six apostles (Predella) 1465–1470	6 Panels	Antoine de Lonhy	Inv. 5 A, B, C, D, E, F
	(Originally panel)		
Disputa di Gesù tra i dottori/ Christ among the doctors 1510	Panel	Defendente Ferrari	343/D
Libro d’Ore all’uso di Chalon-sur-Saône/ Book of Hours in use in Chalon-sur-Saône 1460	Illuminated manuscript	Antoine de Lonhy	inv. 399
Madonna col bambino e santi/ Madonna and Child with Saints 1435	Panel	Guglielmetto Fantini	418/D
Genealogia della Vergine/Holy Kinship 1501–1503	Panel	Gandolfino da Roreto	419/D
Pietà 1465	Mural painting	Collaborator of Antoine de Lonhy	428/D
Madonna col bambino e sant’Anna/ Madonna and Child with saint Anne 1510–1513	Panel	Giovanni Martino Spanzotti	431/D
Sposalizio della Vergine/ Marriage of the Virgin 1504	Panel	Defendente Ferrari	433/D
Sbarco di santa Maria Maddalena a Marsiglia/Arrival of saint Mary Magdalen in Marseille 1505	Panel	Defendente Ferrari	436/D
Santa Caterina/Saint Catherine end of XV century	Panel	Master of Santa Margherita of Crea Chapel	441/D
Incoronazione della Vergine e annunciazione/Coronation of the Virgin and Annunciation 1525–1530	Panel	Defendente Ferrari	442/D
Madonna in trono col bambino; San Giacomo Maggiore; San Giovanni Evangelista; San Giovanni Battista; San Tommaso d’Aquino; due donatori/Madonna enthroned with child; St. James the Greater; Saint John the Evangelist; St. John Baptis; Saint Thomas Aquinas; two donors. 1495	Panel	Macrino d’Alba	448/D
Madonna con bambino; Crocifissione; Sant’Orsola; Sant’Eulalia; San Giovanni Battista; San Giulio/Madonna with child; Crucifixion; Saint Ursula; Saint Eulalia; st. John Baptis; San Giulio 1525	Panel	Pietro Grammorseo	460/D
Presentazione di Gesù al tempio/Presentation of Jesus in the temple 1525–1530	Panel	Defendente Ferrari	462/D
Trinità/Trinity 1465–1470	canvas	Antoine de Lonhy	470/D
Madonna in trono col bambino e quattro angeli/Madonna enthroned with child and four angels 1475–1480	Panel	Giovanni Martino Spanzotti	476/D
Adorazione del bambino/Holy Child 1513	Panel	Gerolamo Giovenone	483/D
Crocifissione/Crucifixion mid XVI century	Panel	Gaudenzio Ferrari	489/D
Adorazione dei magi/Adoration of the Magi 1505	Panel	Gaudenzio Ferrari	491/D
Adorazione del bambino a lume di notte/Holy Child adoration in night light 1510	Panel	Defendente Ferrari	512/D
Noli me tangere 1515–1520	Panel	Sperindio Cagnoli	516/D
Madonna col bambino; Sant’Eusebio; Sant’Apollonia; Sant’Alberto Carmelitano; Santa Caterina; Donatori/Madonna and Child; Saint Eusebius; Saint Apollonia; Saint Albert the Carmelite; Saint Catherine; Donors 1519	Panel	Eusebio Ferrari	721/D
Stimmate di san Francesco; Madonna allattante il Bambino; la Trinità e Santi/Stigmata of Saint Francis; Madonna nursing the Child; the Trinity and Saints end of XIV century	Panel	Pietro d’Alba	774/D
S. Monica 1498	Panel	Maestro di Andriola de Barrachis	787/D
Morte della Vergine/Death of the Virgin 1470	Canvas (Originally panel)	Antoine de Lonhy	No code Balbo Bertone Collection (BBC)

## Data Availability

The data presented in this study are available on request from the corresponding authors.

## Supplementary Materials

The following supporting information can be downloaded at: https://www.mdpi.com/article/10.3390/molecules29246043/s1, Figure S1. Sei apostoli/Six apostles, 1465–1470, Antoine de Lonhy (Museo Civico d’Arte Antica, Inv. 5); Figure S2. Sei apostoli/six apostles (panel A), 1465–1470, Antoine de Lonhy (Museo Civico d’Arte Antica, Inv. 5); Figure S3. Sei apostoli/six apostles (panel B), 1465–1470, Antoine de Lonhy (Museo Civico d’Arte Antica, Inv. 5); Figure S4. Sei apostoli/six apostles (panel C), 1465–1470, Antoine de Lonhy (Museo Civico d’Arte Antica, Inv. 5); Figure S5. Sei apostoli/six apostles (panel D), 1465–1470, Antoine de Lonhy (Museo Civico d’Arte Antica, Inv. 5); Figure S6. Sei apostoli/six apostles (panel E), 1465–1470, Antoine de Lonhy (Museo Civico d’Arte Antica, Inv. 5); Figure S7. Sei apostoli/six apostles (panel F), 1465–1470, Antoine de Lonhy (Museo Civico d’Arte Antica, Inv. 5); Figure S8. Libro d’Ore all’uso di Chalon-sur-Saône/Book of Hours in use in Chalon-sur- Saône, 1460, Antoine de Lonhy (Museo Civico d’Arte Antica, inv. 399, f.1r); Figure S9. Libro d’Ore all’uso di Chalon-sur-Saône/Book of Hours in use in Chalon-sur- Saône, 1460, Antoine de Lonhy (Museo Civico d’Arte Antica, inv. 399, f. 49v); Figure S10. Libro d’Ore all’uso di Chalon-sur-Saône/Book of Hours in use in Chalon-sur- Saône, 1460, Antoine de Lonhy (Museo Civico d’Arte Antica, inv. 399, f. 31v); Figure S11. Disputa di Gesù tra i dottori/Christ among the doctors, 1510, Defendente Ferrari (Museo Civico d’Arte Antica, 343/D); Figure S12. Madonna col bambino e santi/Madonna and Child with Saints, 1435, Guglielmetto Fantini (Museo Civico d’Arte Antica, 418/D); Figure S13. Genealogia della Vergine/Holy Kinship, 1501–1503, Gandolfino da Roreto (Museo Civico d’Arte Antica, 419/D); Figure S14. Pietà, 1465, collaborator of Antoine de Lonhy (Museo Civico d’Arte Antica, 428/D); Figure S15. Madonna col bambino e sant’Anna/Madonna and Child with saint Anne, 1510–1513, Giovanni Martino Spanzotti (Museo Civico d’Arte Antica, 431/D); Figure S16. Sposalizio della Vergine/Marriage of the Virgin, 1504, Defendente Ferrari (Museo Civico d’Arte Antica, 433/D); Figure S17. Sbarco di santa Maria Maddalena a Marsiglia/Arrival of saint Mary Magdalen in Marseille, 1505, Defendente Ferrari (Museo Civico d’Arte Antica, 436/D); Figure S18. Santa Caterina/Saint Catherine, end of XV century, Master of Santa Margherita of Crea Chapel (Museo Civico d’Arte Antica, 441/D); Figure S19. Incoronazione della Vergine e annunciazione/Coronation of the Virgin and Annunciation, 1525–1530, Defendente Ferrari (Museo Civico d’Arte Antica, 442/D); Figure S20. Madonna in trono col bambino; San Giacomo Maggiore; San Giovanni Evangelista; San Giovanni Battista; San Tommaso d’Aquino; due donatori/Madonna enthroned with child; St. James the Greater; Saint John the Evangelist; St. John Baptis; Saint Thomas Aquinas; two donors., 1495, Macrino d’Alba (Museo Civico d’Arte Antica, 448/D); Figure S21. Madonna con bambino; Crocifissione; Sant’Orsola; Sant’Eulalia; San Giovanni Battista; San Giulio/Madonna with child; Crucifixion; Saint Ursula; Saint Eulalia; st. John Baptis; San Giulio, 1525, Pietro Grammorseo (Museo Civico d’Arte Antica, 460/D); Figure S22. Presentazione di Gesù al tempio/Presentation of Jesus in the temple, 1525–1530, Defendente Ferrari (Museo Civico d’Arte Antica, 462/D); Figure S23. Trinità/Trinity, 1465–1470, Antoine de Lonhy (Museo Civico d’Arte Antica, 470/D); Figure S24. Madonna in trono col bambino e quattro angeli/Madonna enthroned with child and four angels, 1475–1480, Giovanni Martino Spanzotti (Museo Civico d’Arte Antica, 476/D); Figure S25. Adorazione del bambino/Holy Child, 1513, Gerolamo Giovenone (Museo Civico d’Arte Antica, 483/D); Figure S26. Crocifissione/Crucifixion, mid XVI century, Gaudenzio Ferrari (Museo Civico d’Arte Antica, 489/D); Figure S27. Adorazione dei magi/Adoration of the Magi, 1505, Gaudenzio Ferrari (Museo Civico d’Arte Antica, 491/D); Figure S28. Adorazione del bambino a lume di notte/Holy Child adoration in night light, 1510, Defendente Ferrari (Museo Civico d’Arte Antica, 512/D); Figure S29. Noli me tangere, 1515–1520, Sperindio Cagnoli (Museo Civico d’Arte Antica, 516/D); Figure S30. Madonna col bambino; Sant’Eusebio; Sant’Apollonia; Sant’Alberto Carmelitano; Santa Caterina; Donatori/Madonna and Child; Saint Eusebius; Saint Apollonia; Saint Albert the Carmelite; Saint Catherine; Donors, 1519, Eusebio Ferrari (Museo Civico d’Arte Antica, 721/D); Figure S31. Stimmate di san Francesco; Madonna allattante il Bambino; la Trinità e Santi/Stigmata of Saint Francis; Madonna nursing the Child; the Trinity and Saints, end of XIV century, Pietro d’Alba (Museo Civico d’Arte Antica, 774/D); Figure S32. S. Monica, 1498, Maestro di Andriola de Barrachis (Museo Civico d’Arte Antica, 787/D); Figure S33. Morte della Vergine/Death of the Virgin, 1470, Antoine de Lonhy (Balbo Bertone Collection).
