# Supplementary figures and images for: Multi-Analytical and Non-Invasive Approach for Characterising Blackened Areas of Originally Blue Paints
Source: Molecules. 2024 Dec 22;29(24):6043. doi: 10.3390/molecules29246043 (PMC11678357; doi:10.3390/molecules29246043)

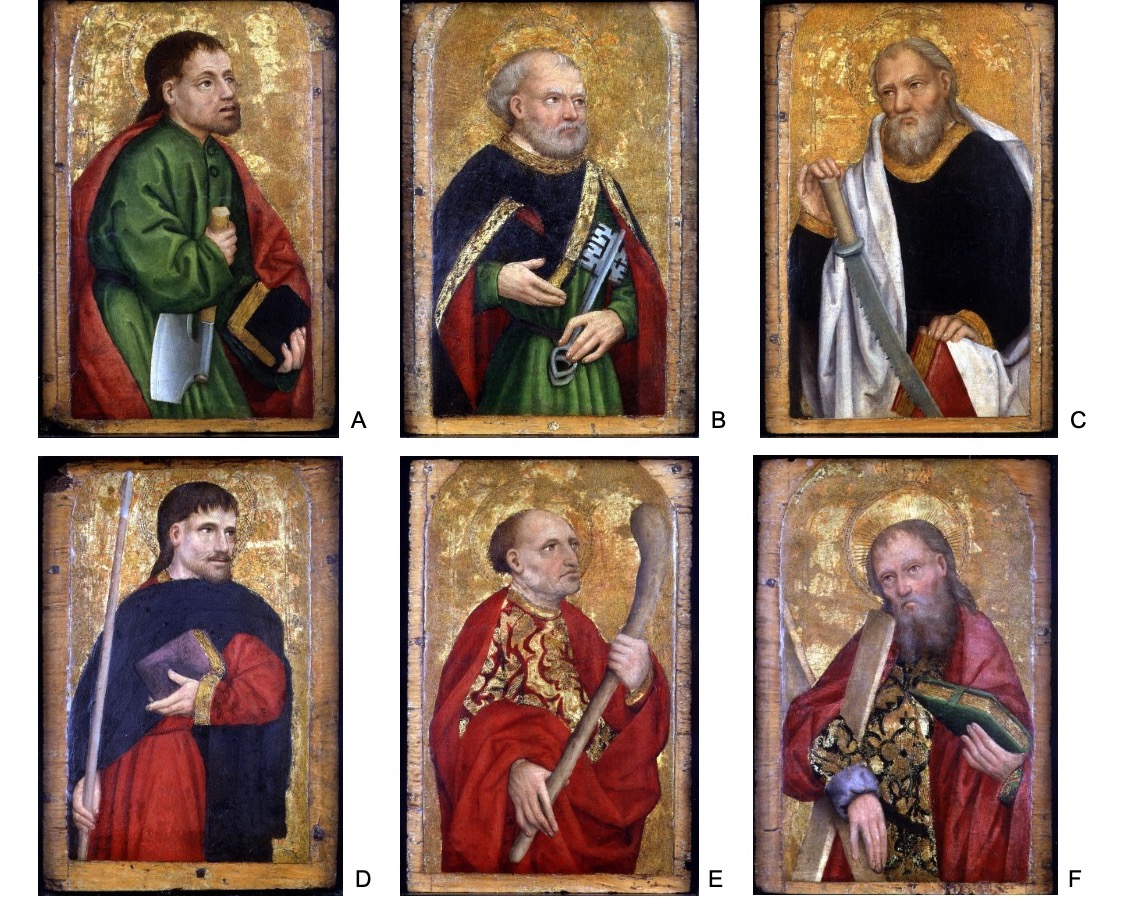

Supplement: Supplementary file 1 [file molecules-29-06043-s001.zip › Figure S1.jpg]

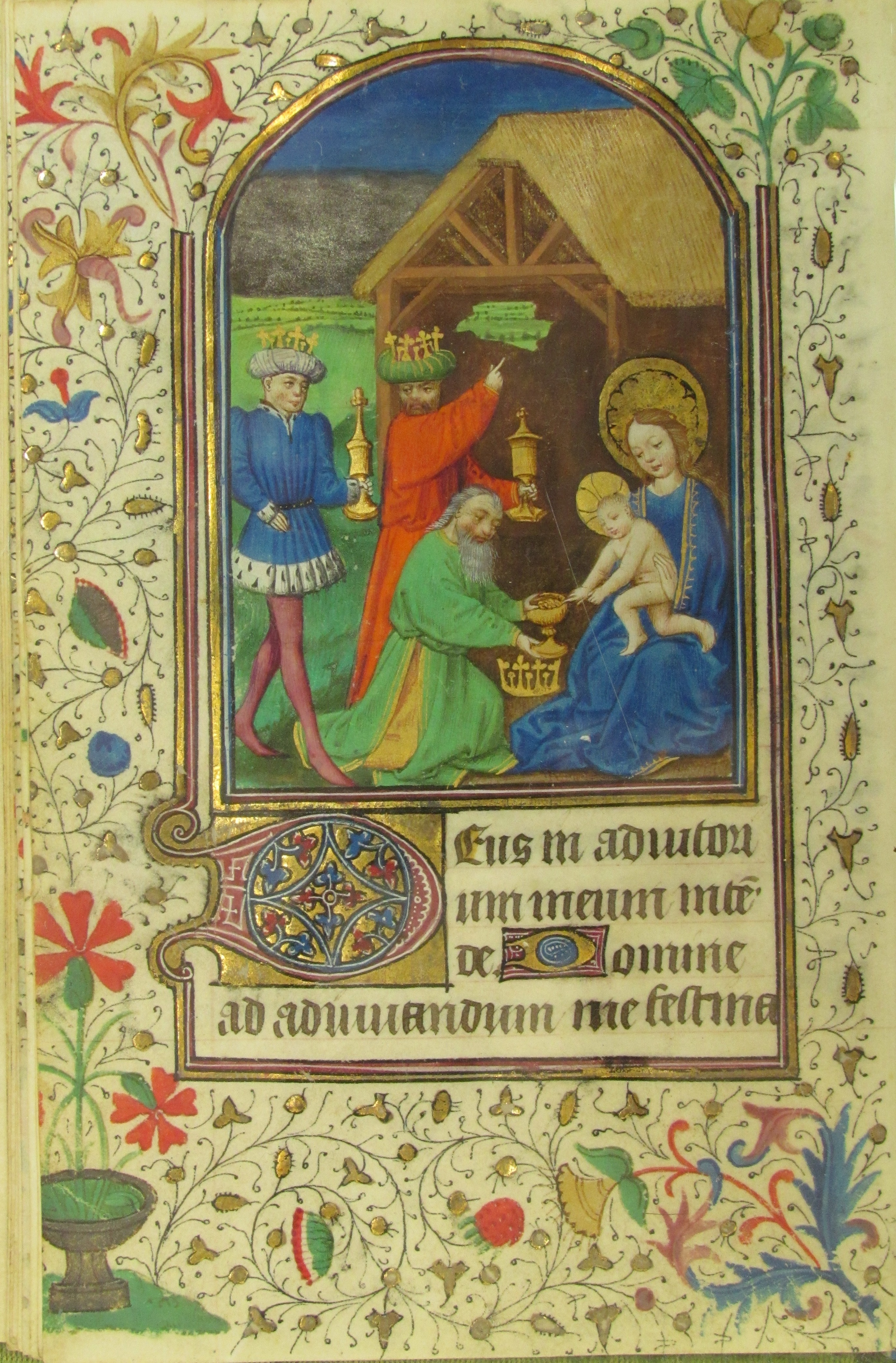

Supplement: Supplementary file 1 [file molecules-29-06043-s001.zip › Figure S10.JPG]

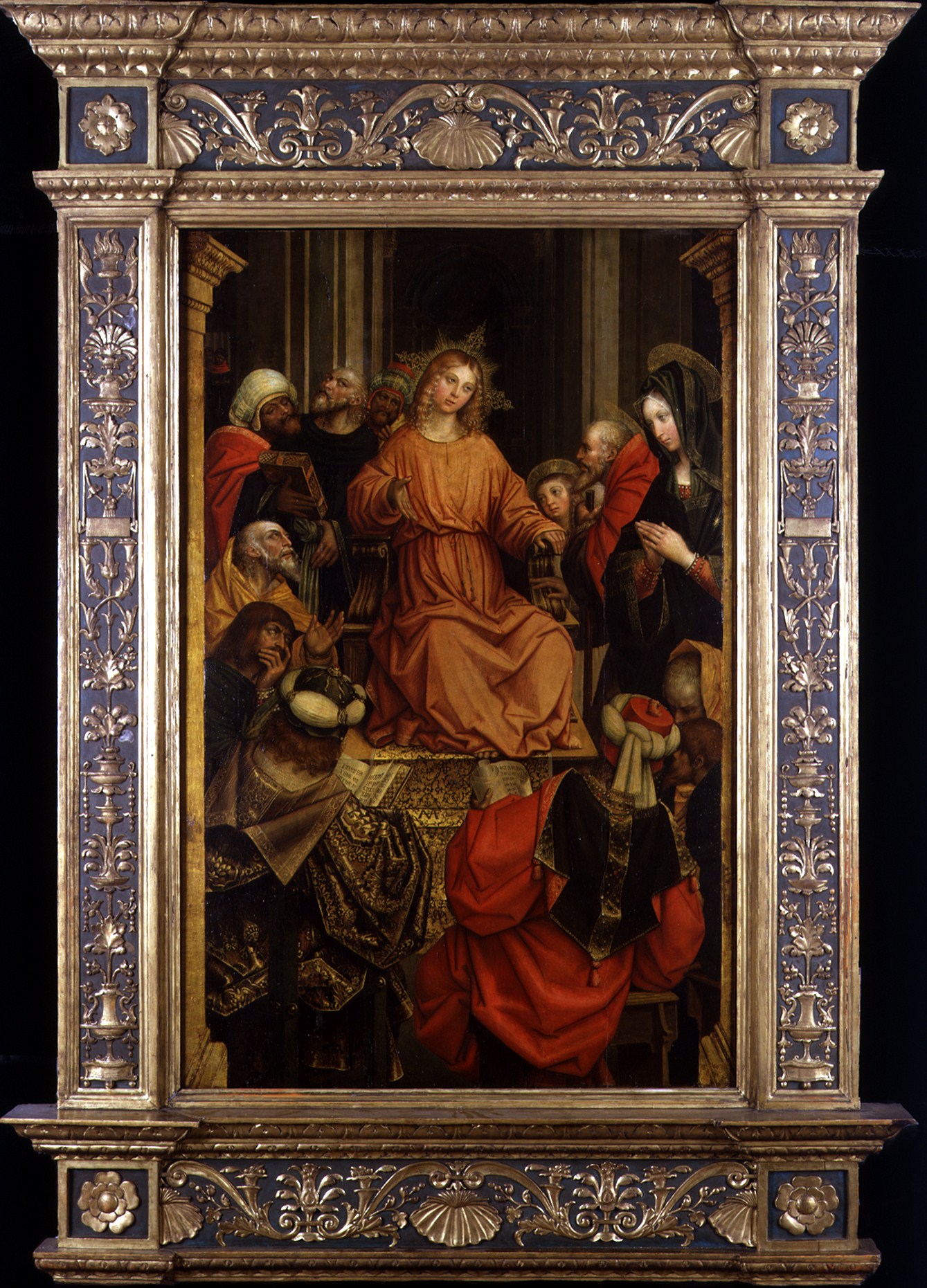

Supplement: Supplementary file 1 [file molecules-29-06043-s001.zip › Figure S11.jpg]

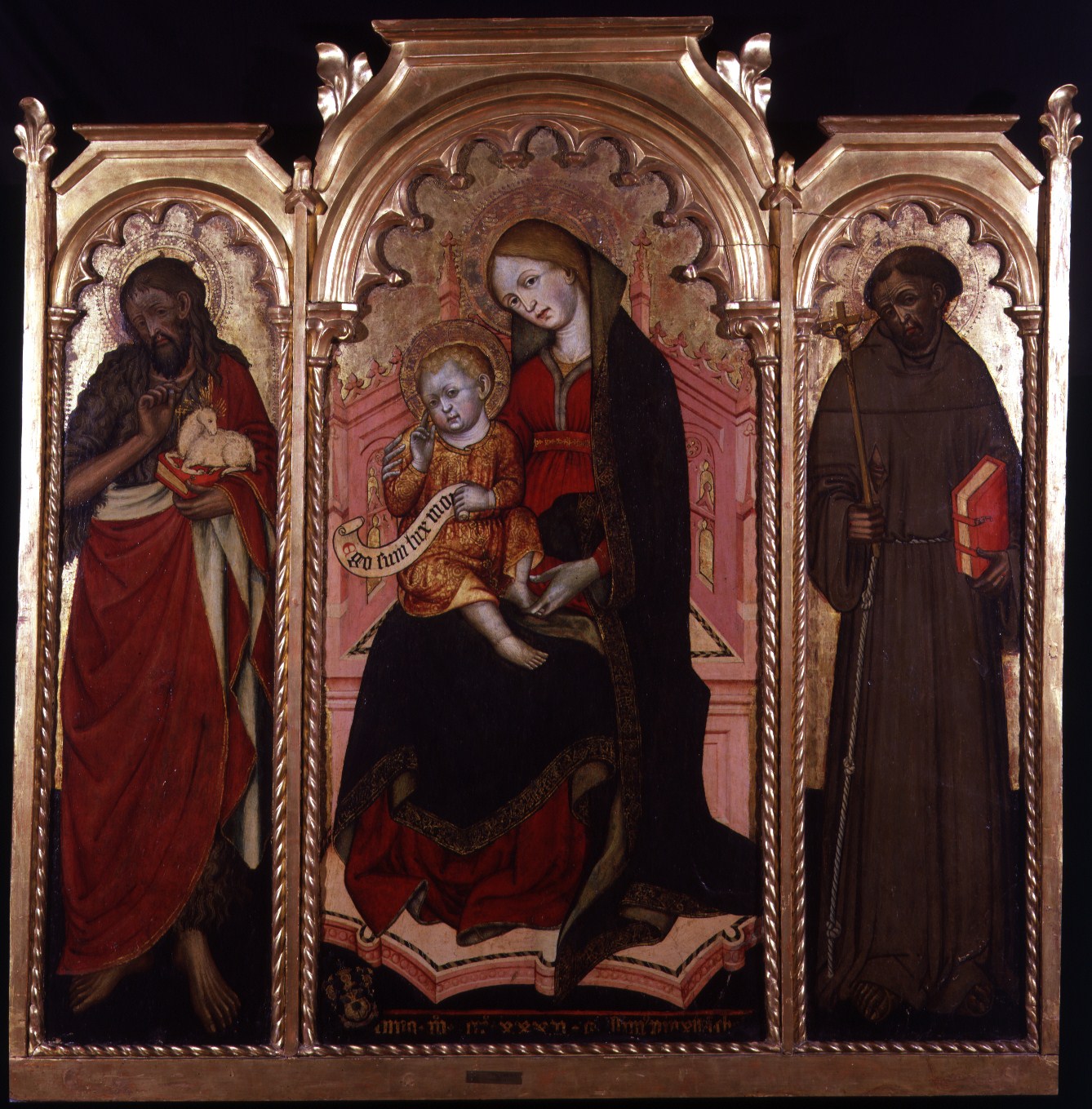

Supplement: Supplementary file 1 [file molecules-29-06043-s001.zip › Figure S12.jpg]

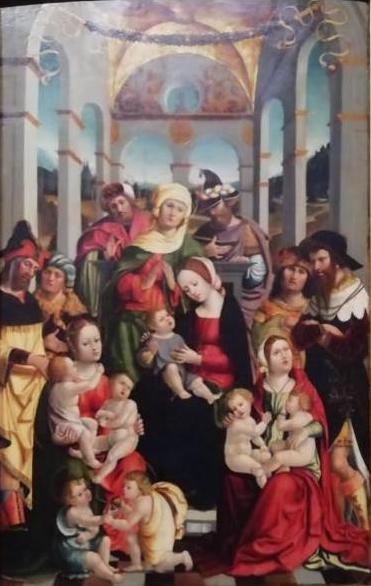

Supplement: Supplementary file 1 [file molecules-29-06043-s001.zip › Figure S13.jpg]

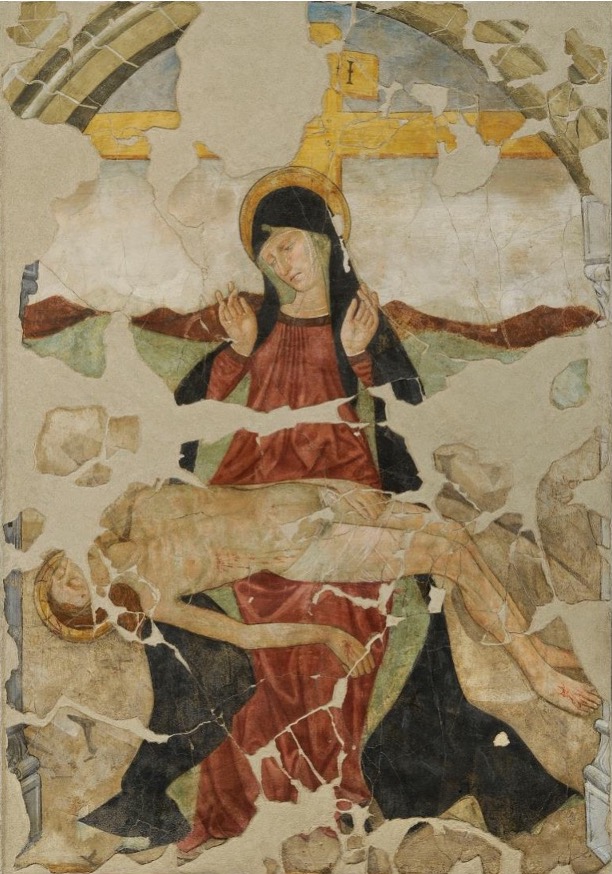

Supplement: Supplementary file 1 [file molecules-29-06043-s001.zip › Figure S14.jpeg]

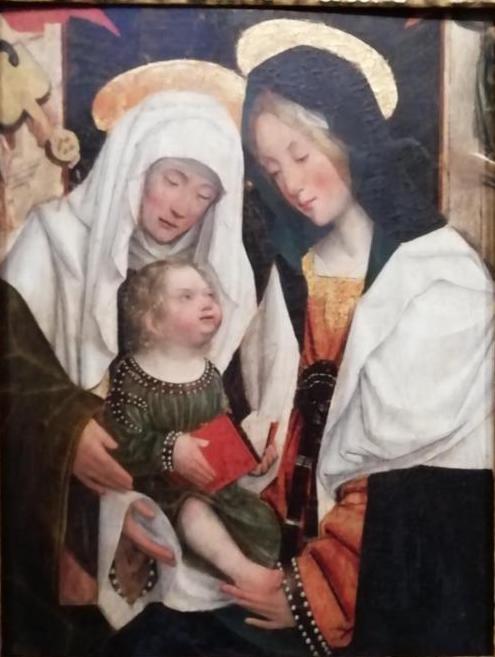

Supplement: Supplementary file 1 [file molecules-29-06043-s001.zip › Figure S15.jpg]

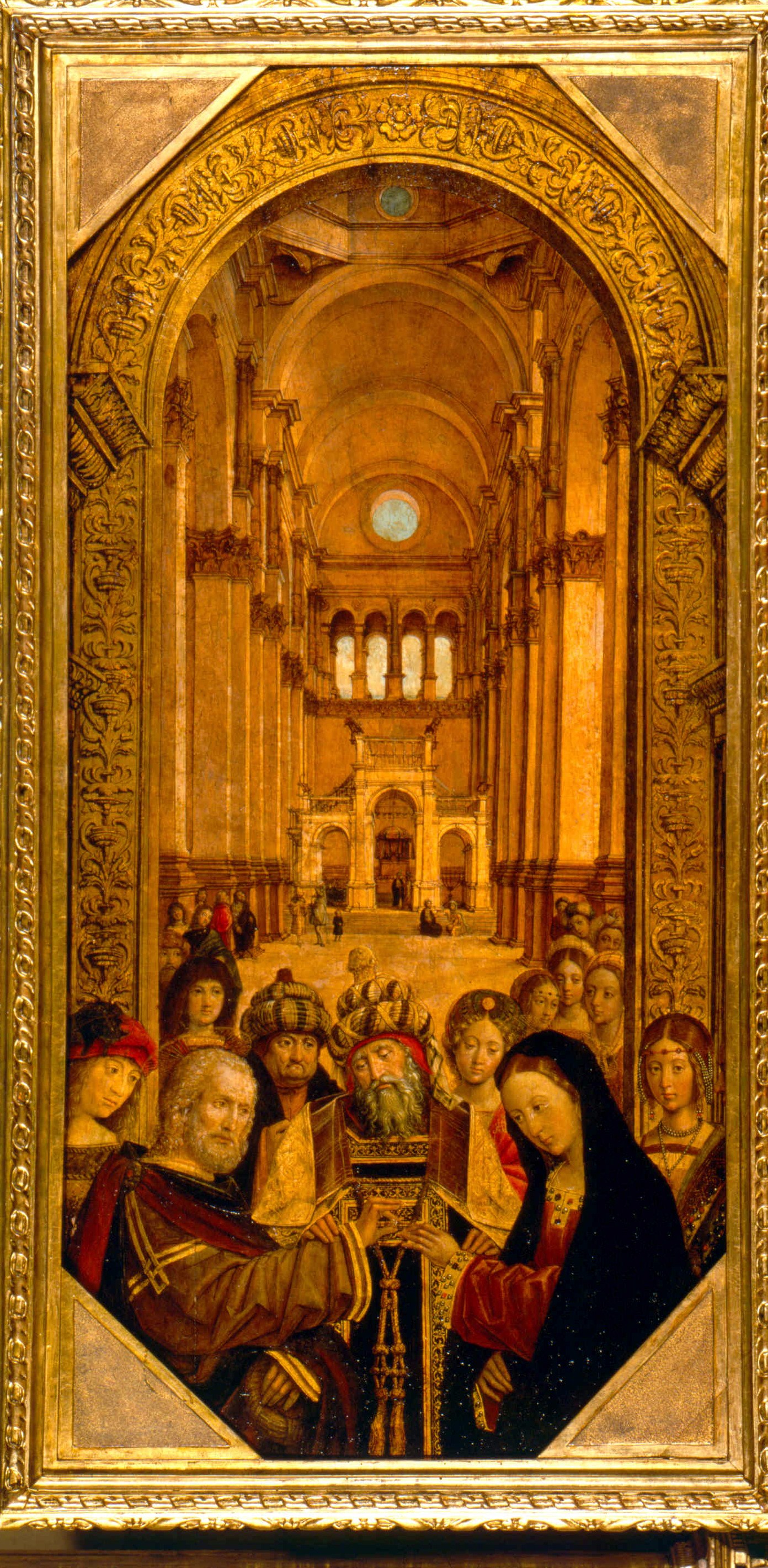

Supplement: Supplementary file 1 [file molecules-29-06043-s001.zip › Figure S16.jpg]

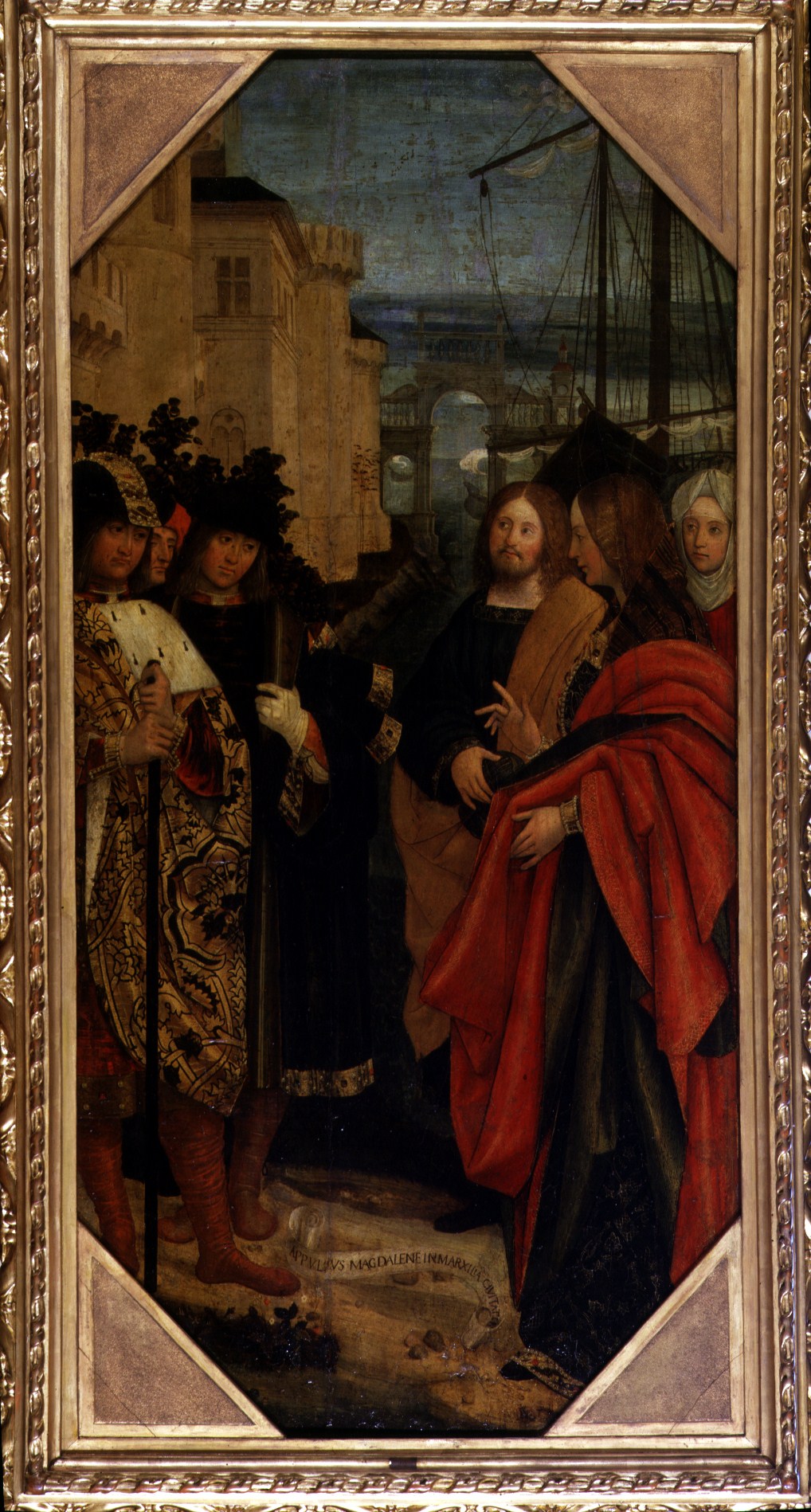

Supplement: Supplementary file 1 [file molecules-29-06043-s001.zip › Figure S17.jpg]

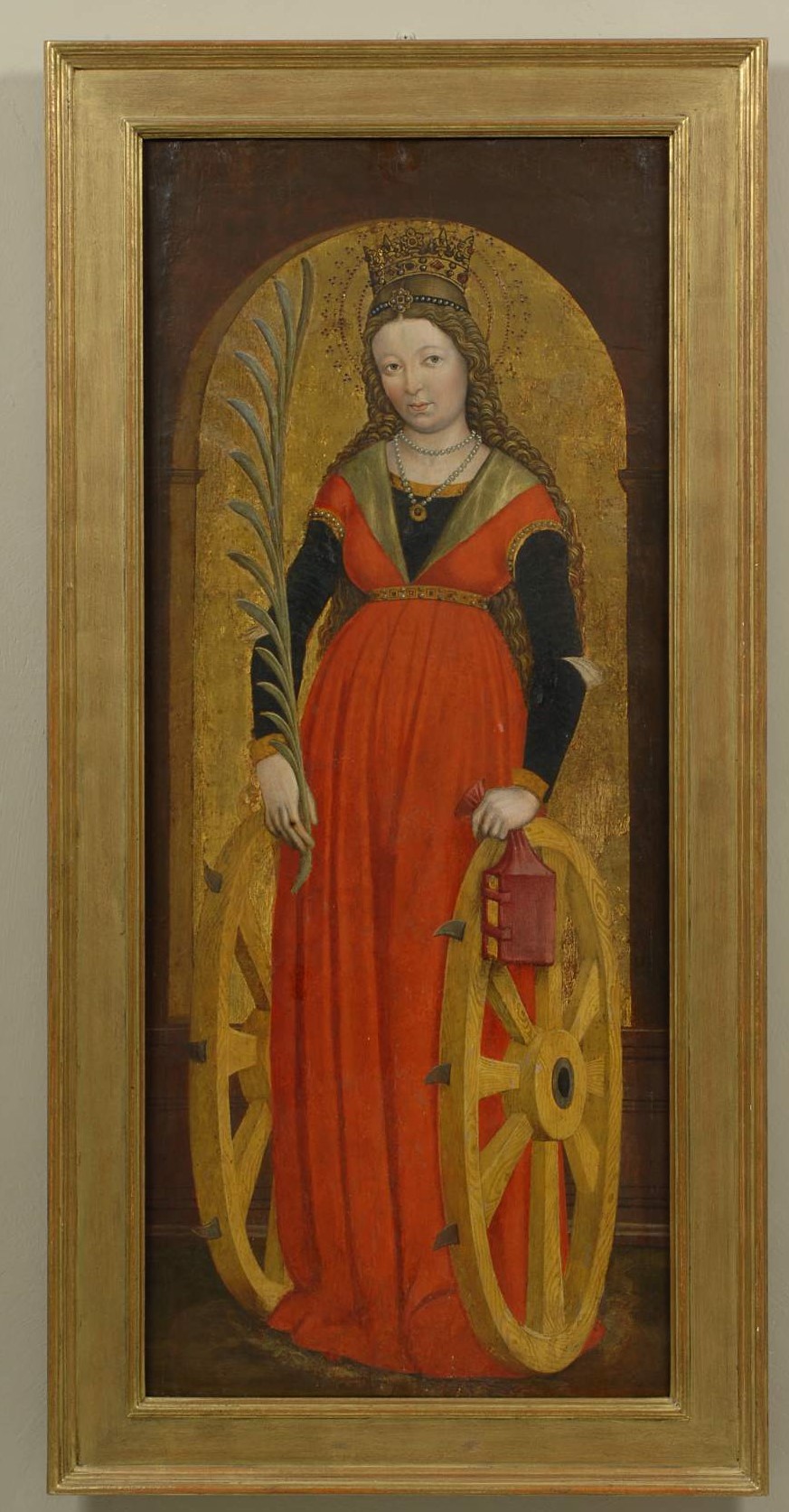

Supplement: Supplementary file 1 [file molecules-29-06043-s001.zip › Figure S18.jpg]

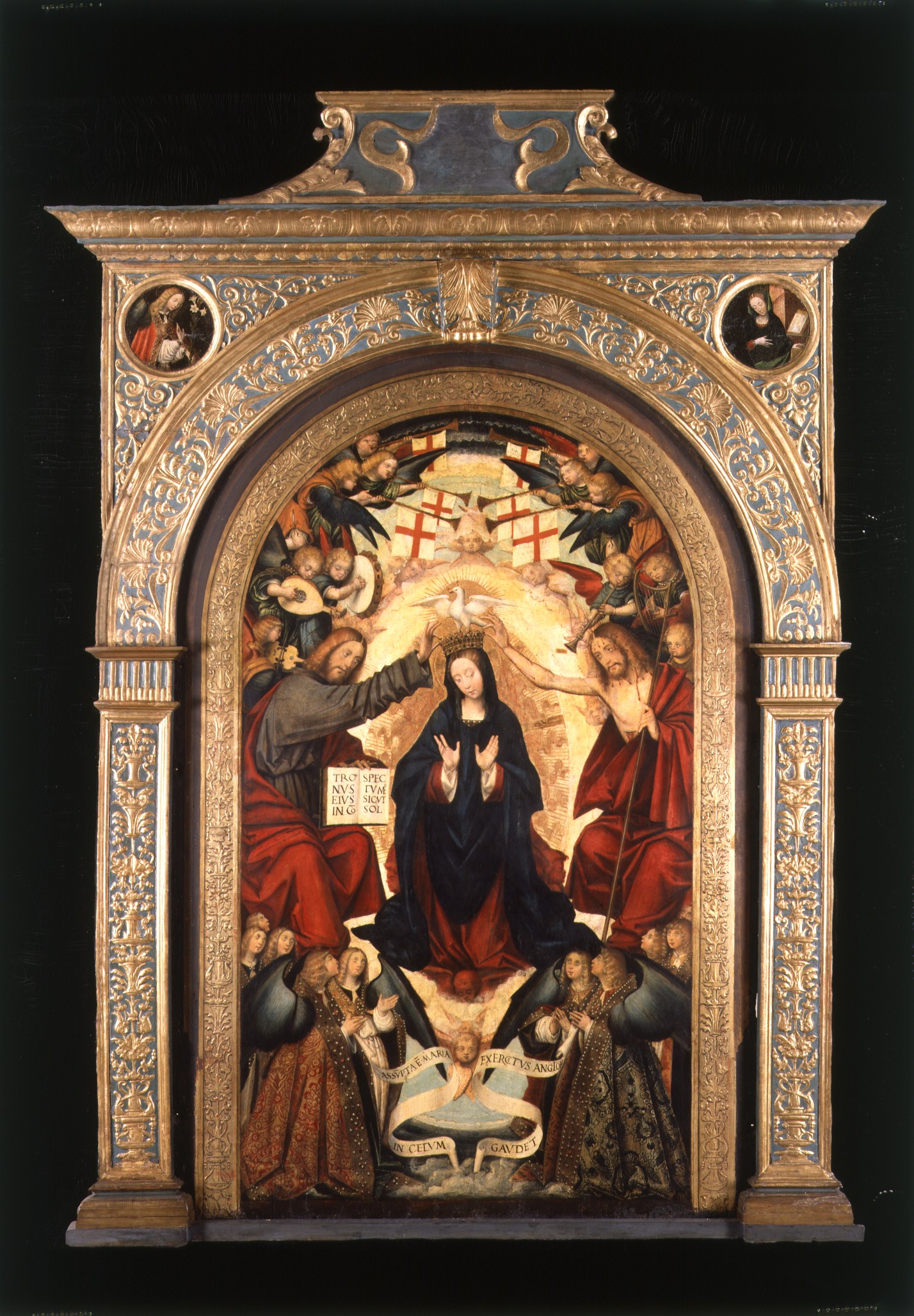

Supplement: Supplementary file 1 [file molecules-29-06043-s001.zip › Figure S19.jpg]

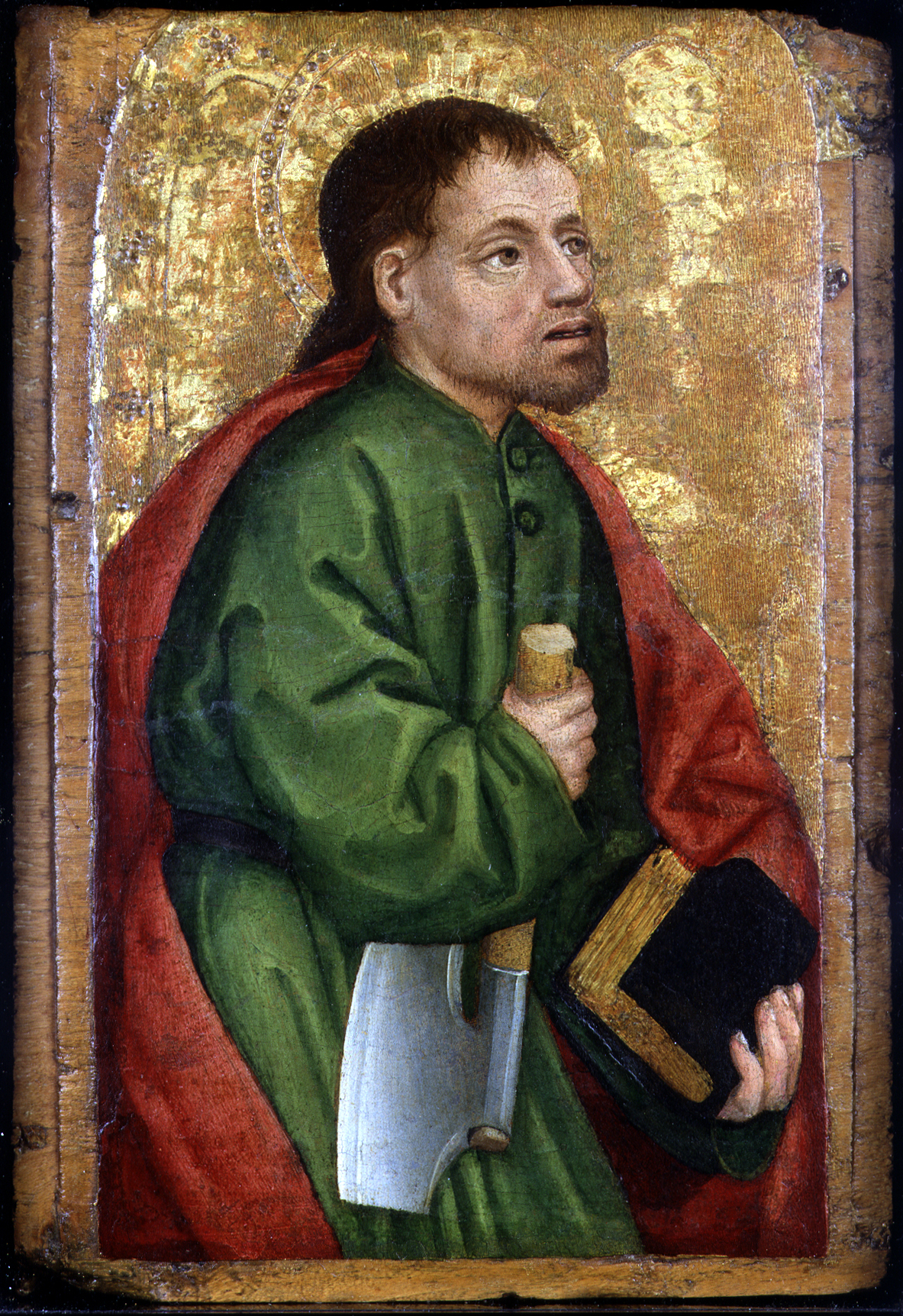

Supplement: Supplementary file 1 [file molecules-29-06043-s001.zip › Figure S2.jpg]

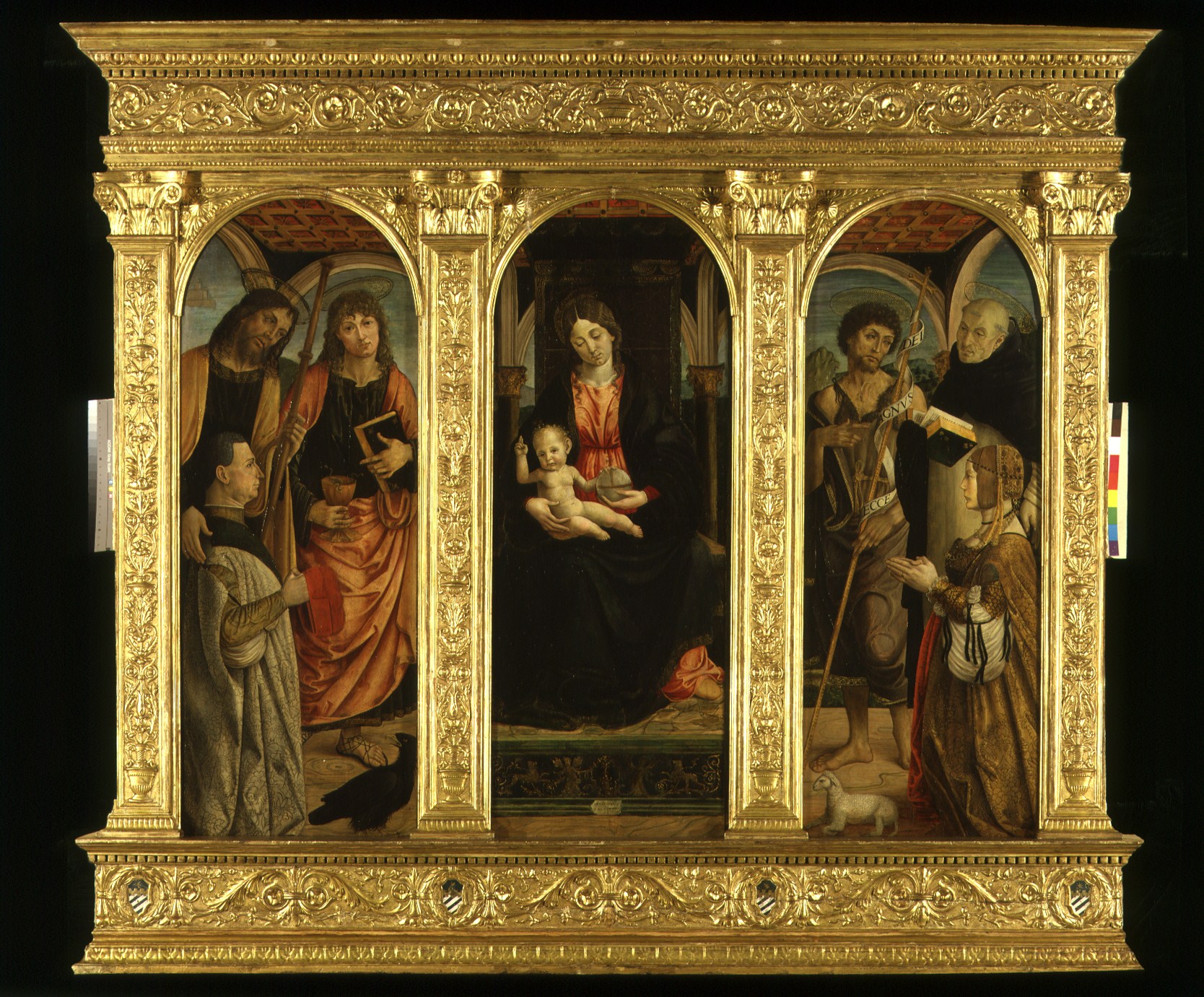

Supplement: Supplementary file 1 [file molecules-29-06043-s001.zip › Figure S20.jpg]

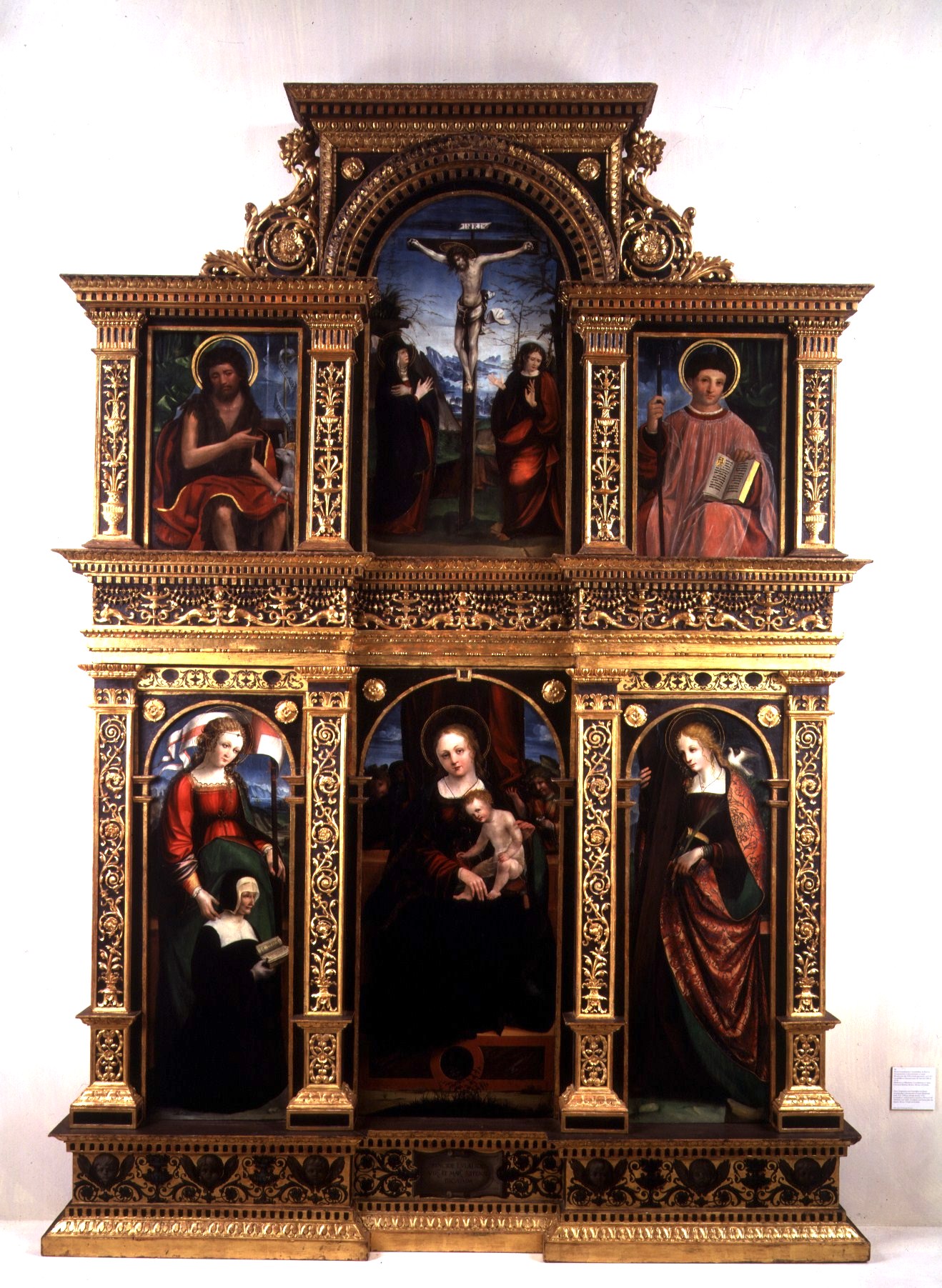

Supplement: Supplementary file 1 [file molecules-29-06043-s001.zip › Figure S21.jpg]

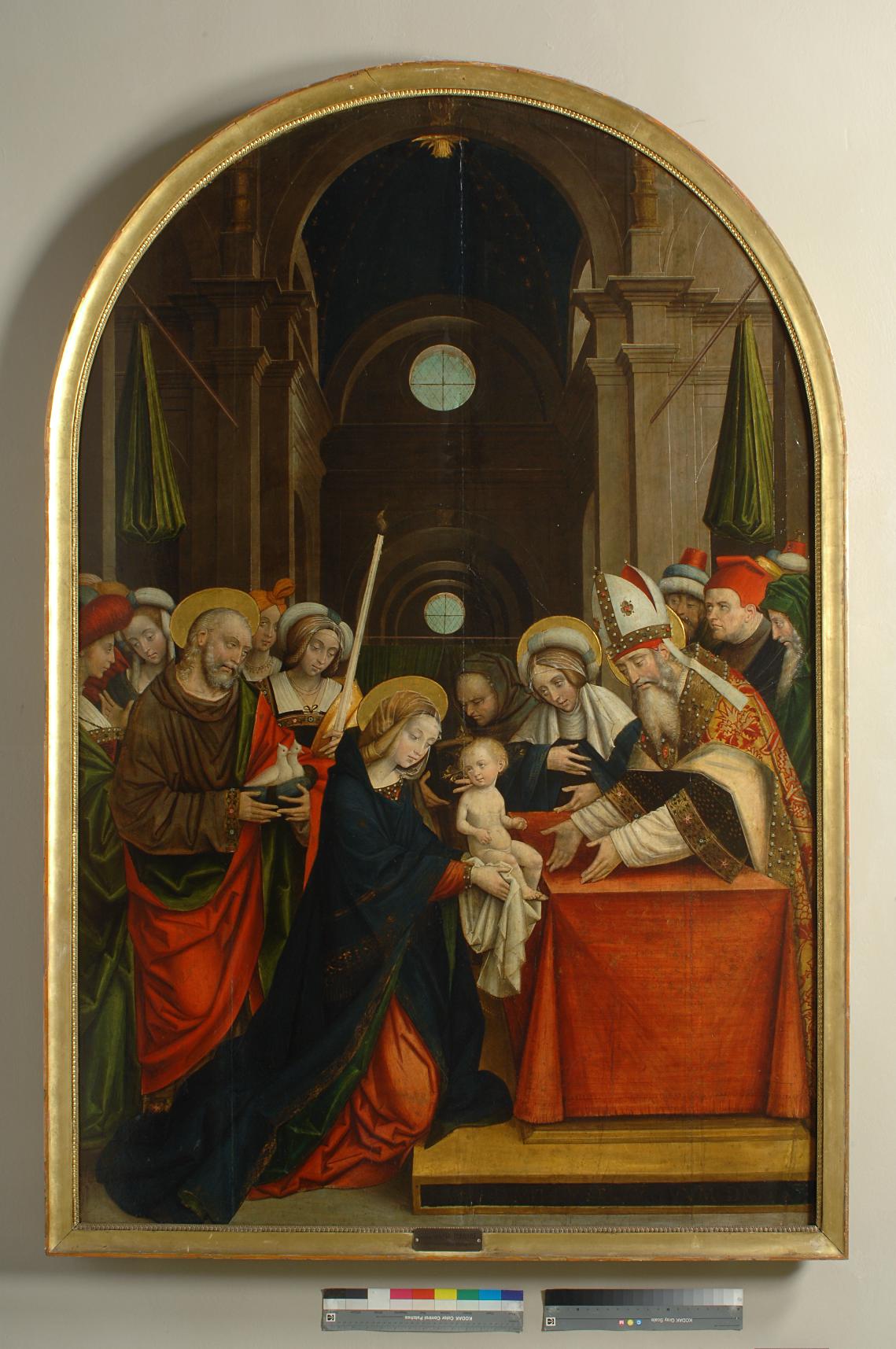

Supplement: Supplementary file 1 [file molecules-29-06043-s001.zip › Figure S22.jpg]

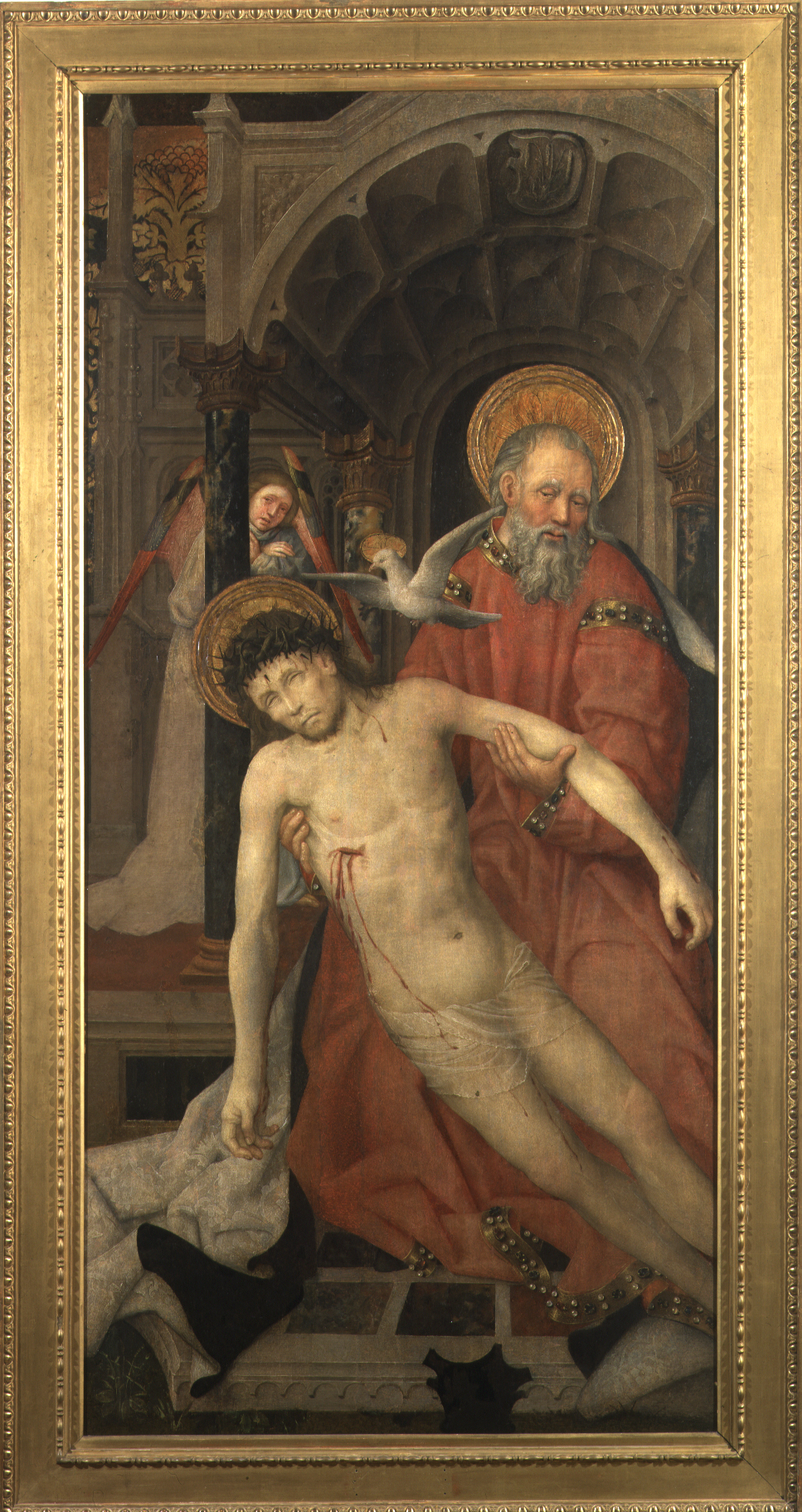

Supplement: Supplementary file 1 [file molecules-29-06043-s001.zip › Figure S23.jpg]

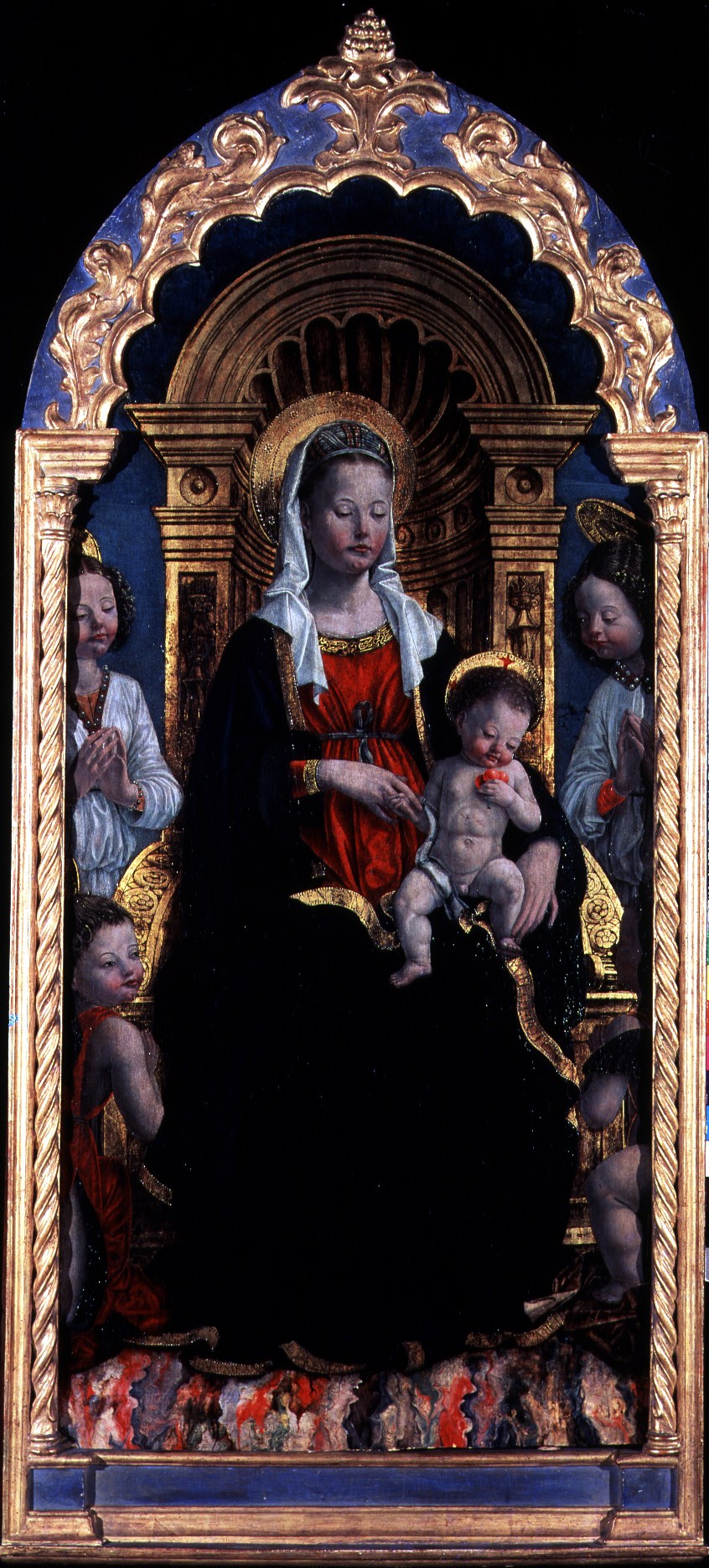

Supplement: Supplementary file 1 [file molecules-29-06043-s001.zip › Figure S24.jpg]

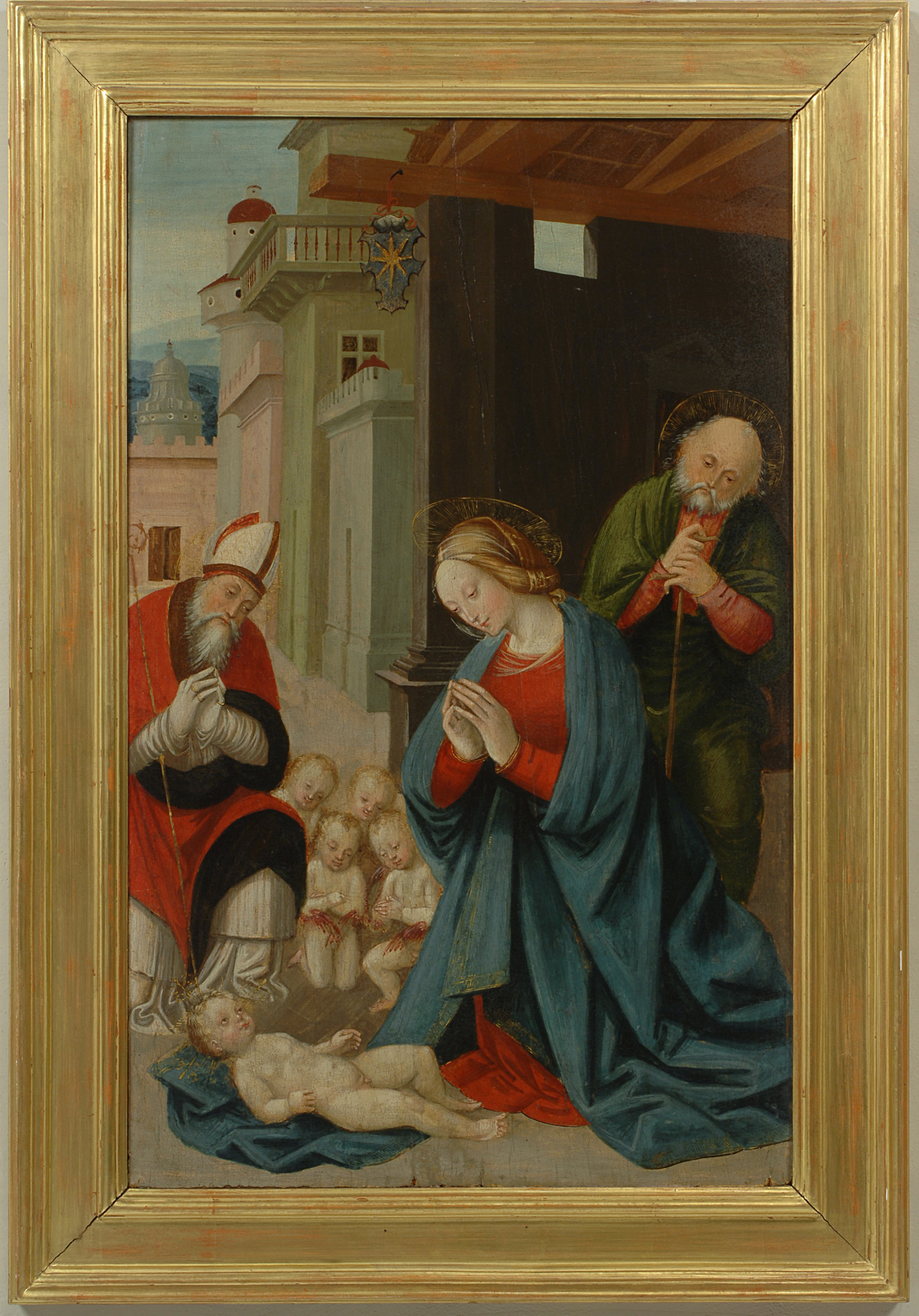

Supplement: Supplementary file 1 [file molecules-29-06043-s001.zip › Figure S25.jpg]

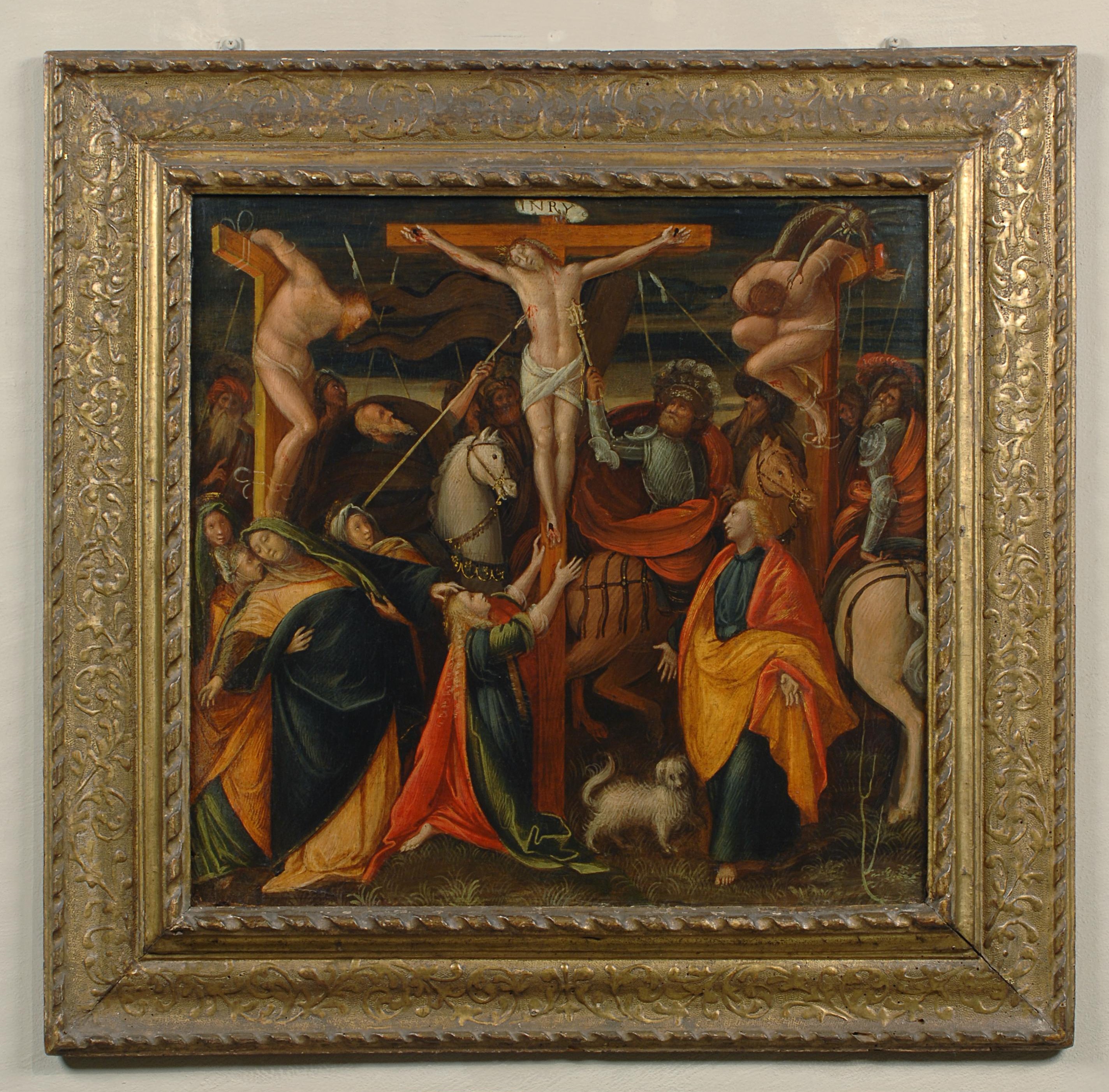

Supplement: Supplementary file 1 [file molecules-29-06043-s001.zip › Figure S26.jpg]

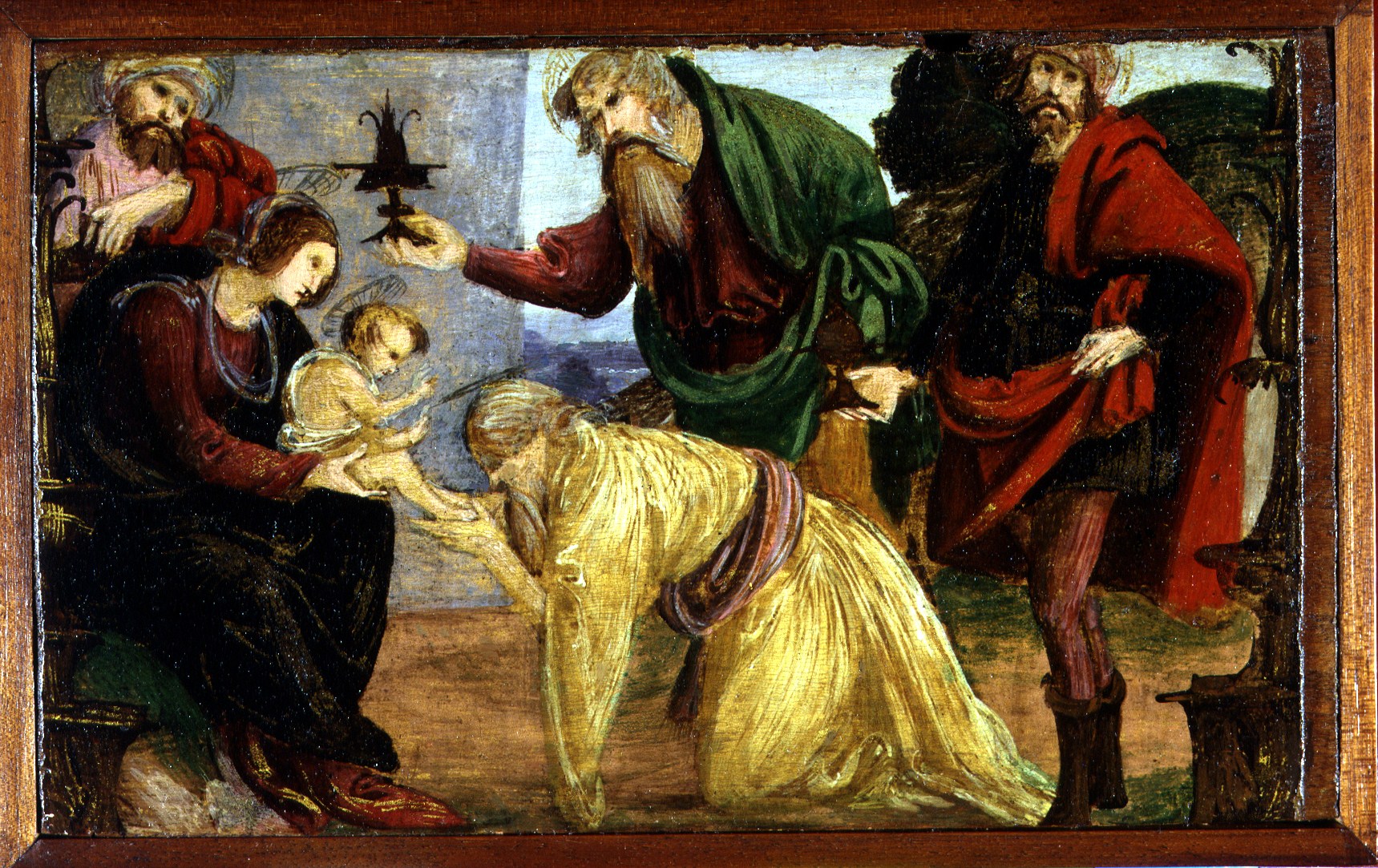

Supplement: Supplementary file 1 [file molecules-29-06043-s001.zip › Figure S27.jpg]

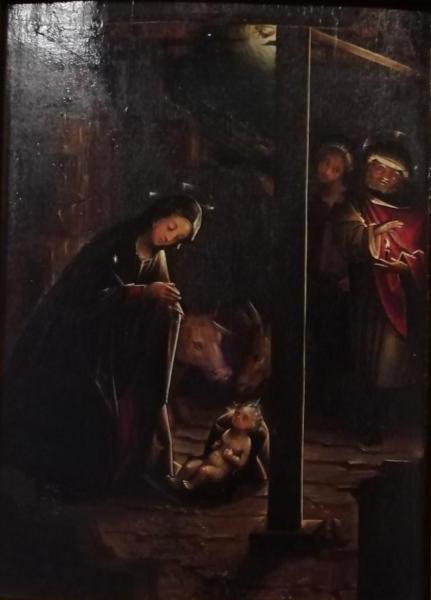

Supplement: Supplementary file 1 [file molecules-29-06043-s001.zip › Figure S28.jpg]

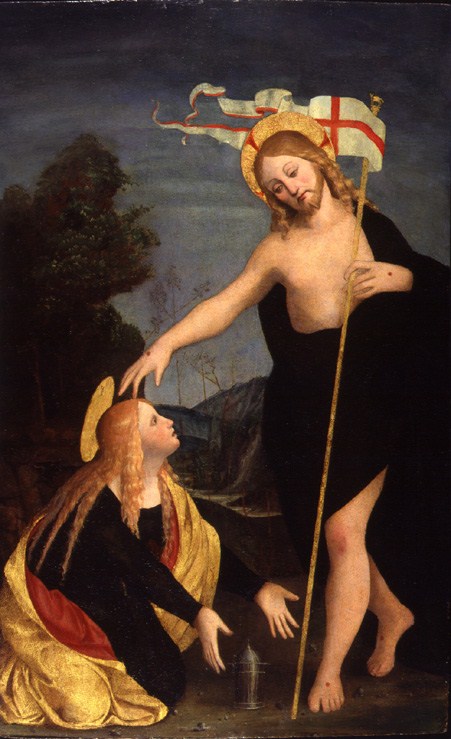

Supplement: Supplementary file 1 [file molecules-29-06043-s001.zip › Figure S29.jpg]

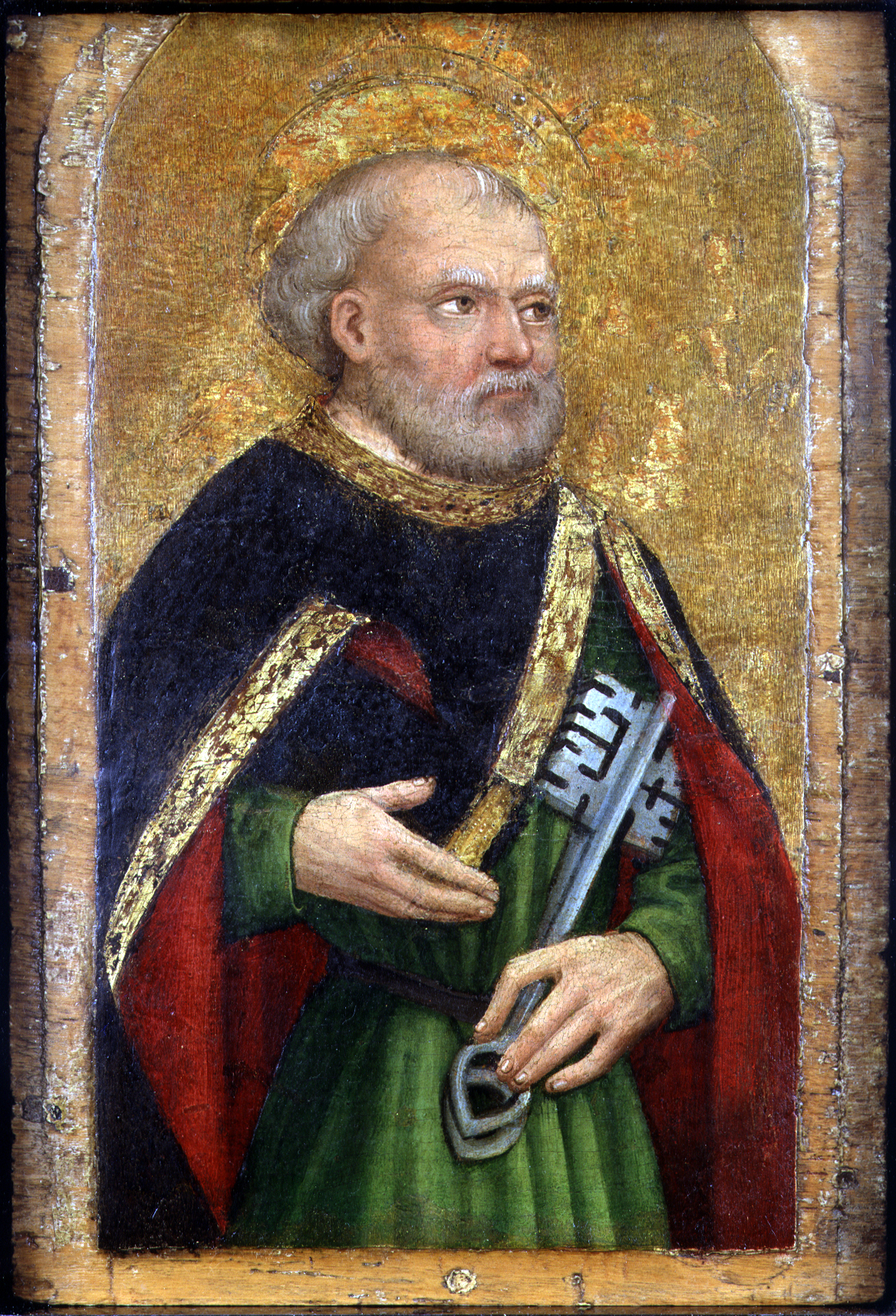

Supplement: Supplementary file 1 [file molecules-29-06043-s001.zip › Figure S3.jpg]

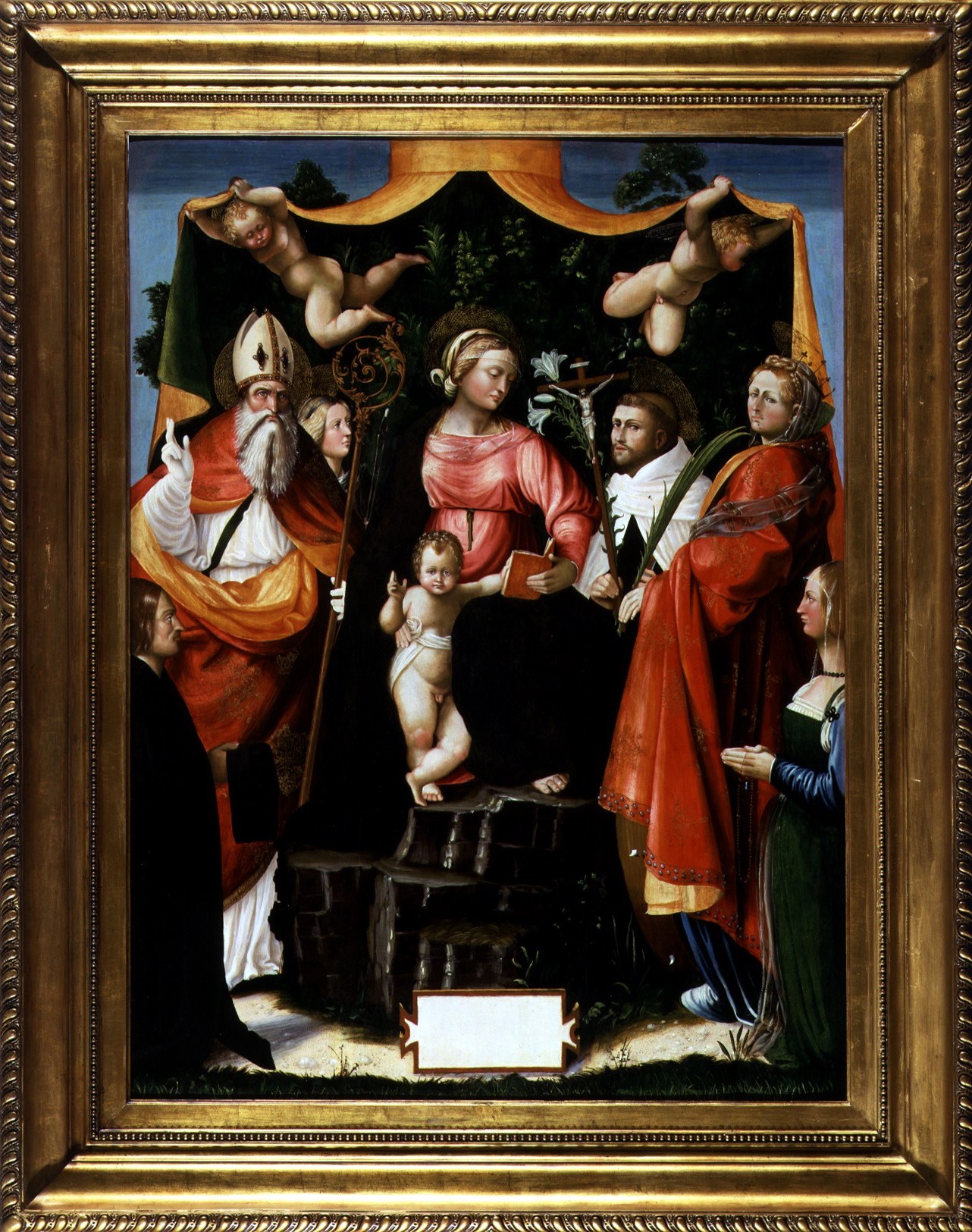

Supplement: Supplementary file 1 [file molecules-29-06043-s001.zip › Figure S30.jpg]

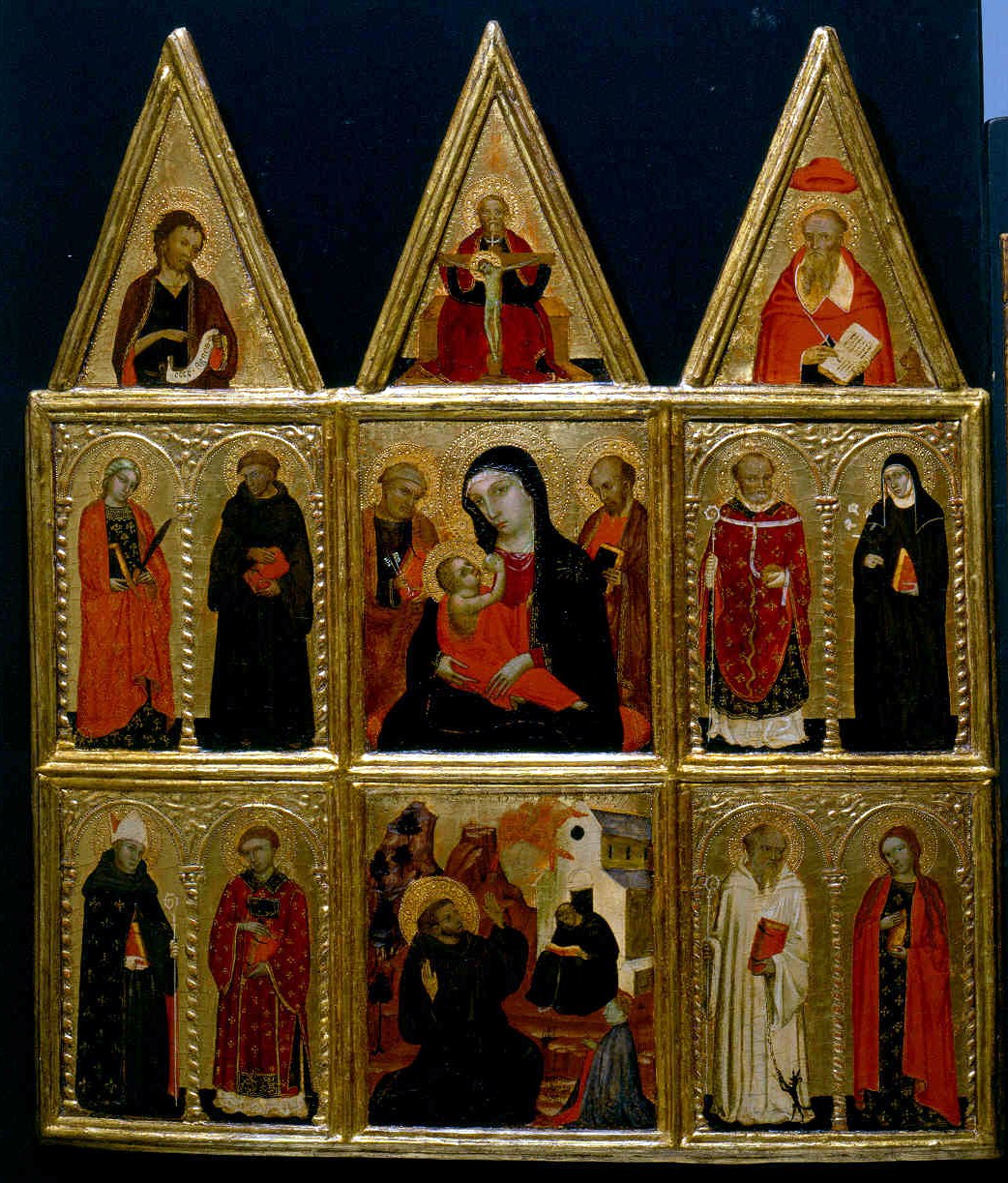

Supplement: Supplementary file 1 [file molecules-29-06043-s001.zip › Figure S31.jpg]

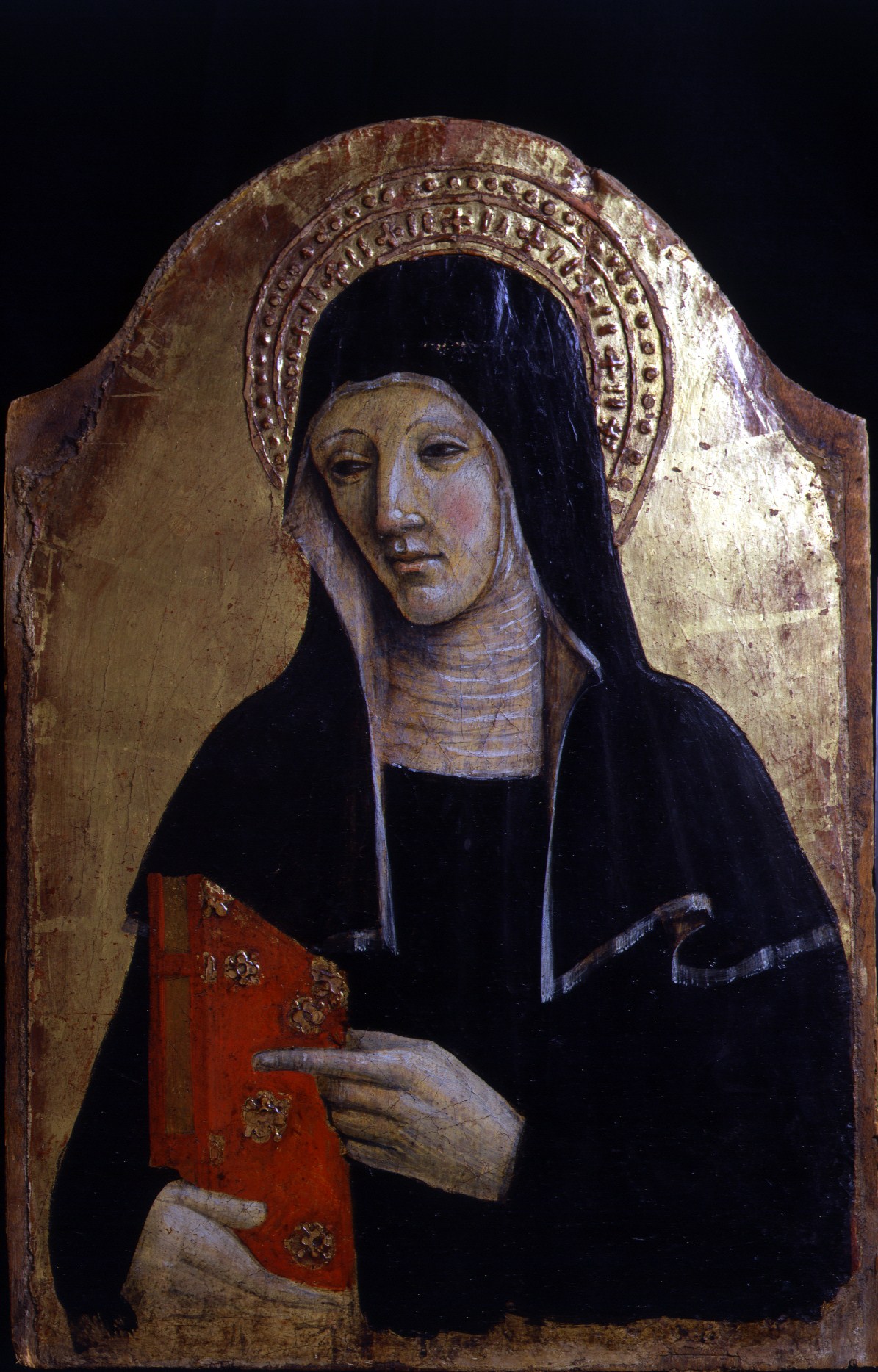

Supplement: Supplementary file 1 [file molecules-29-06043-s001.zip › Figure S32.jpg]

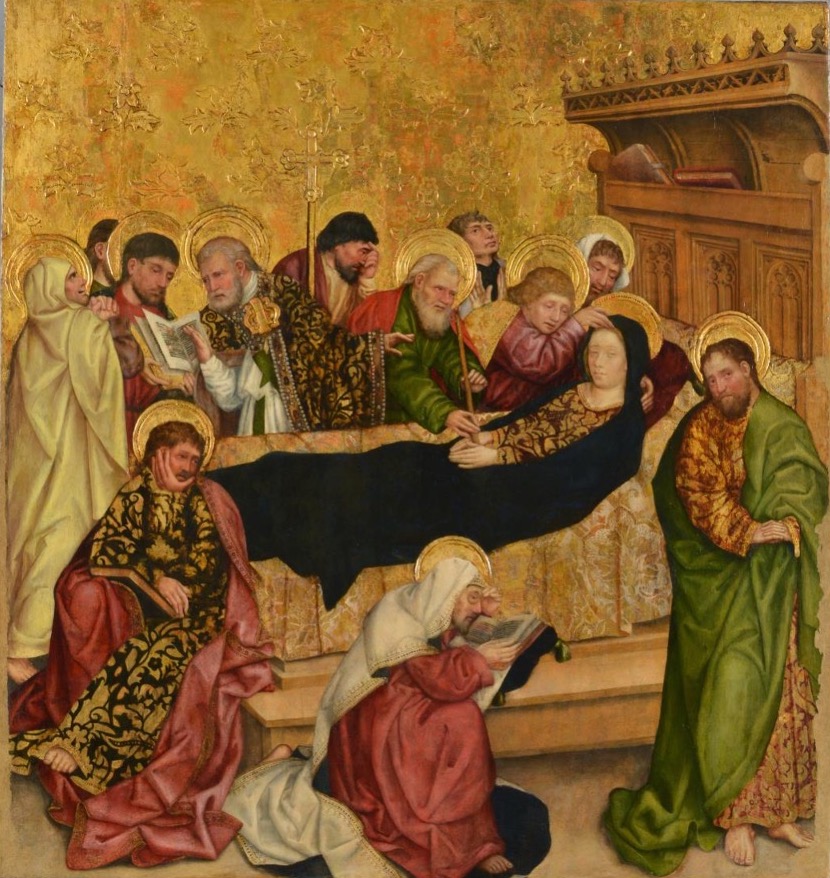

Supplement: Supplementary file 1 [file molecules-29-06043-s001.zip › Figure S33.jpeg]

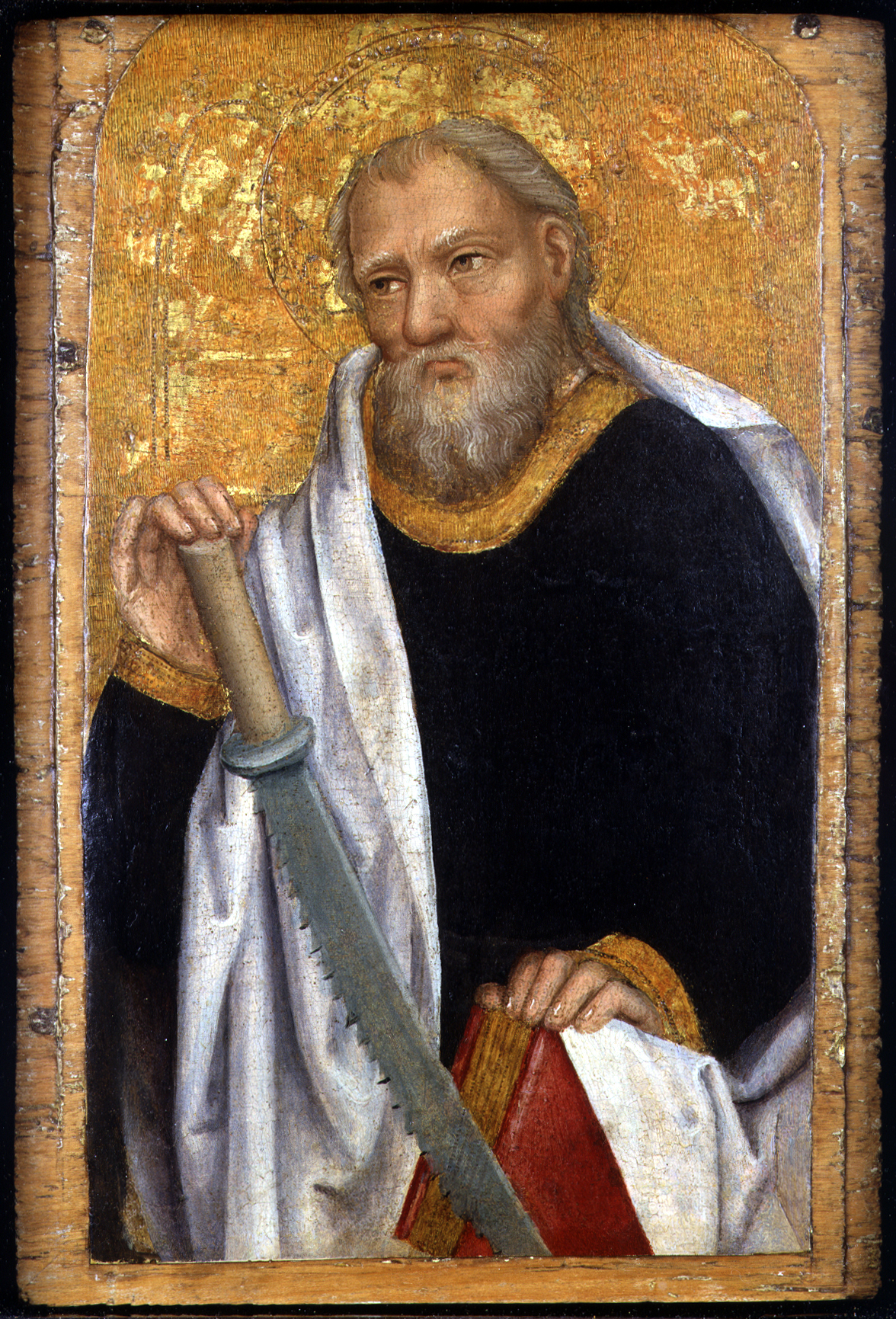

Supplement: Supplementary file 1 [file molecules-29-06043-s001.zip › Figure S4.jpg]

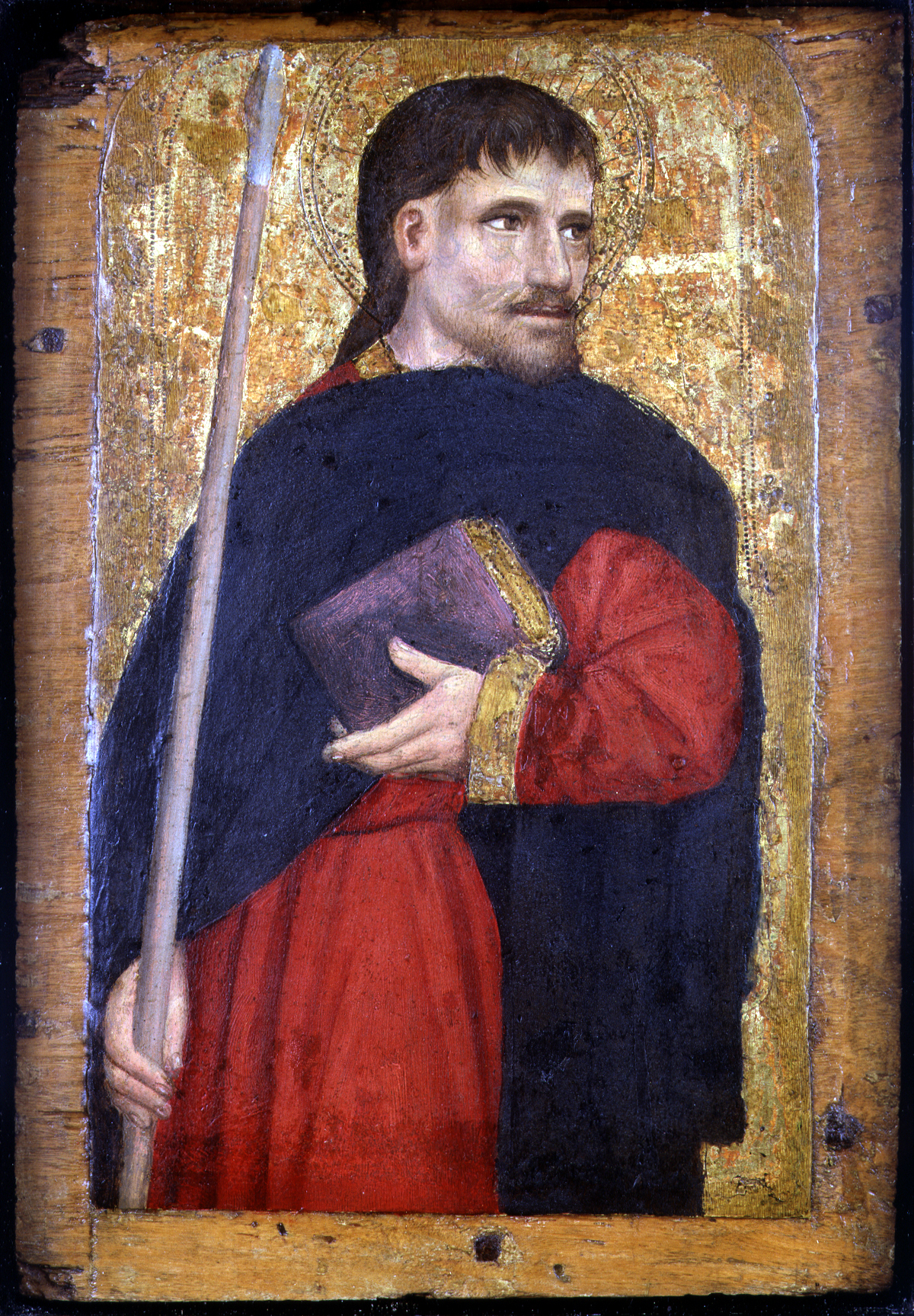

Supplement: Supplementary file 1 [file molecules-29-06043-s001.zip › Figure S5.jpg]

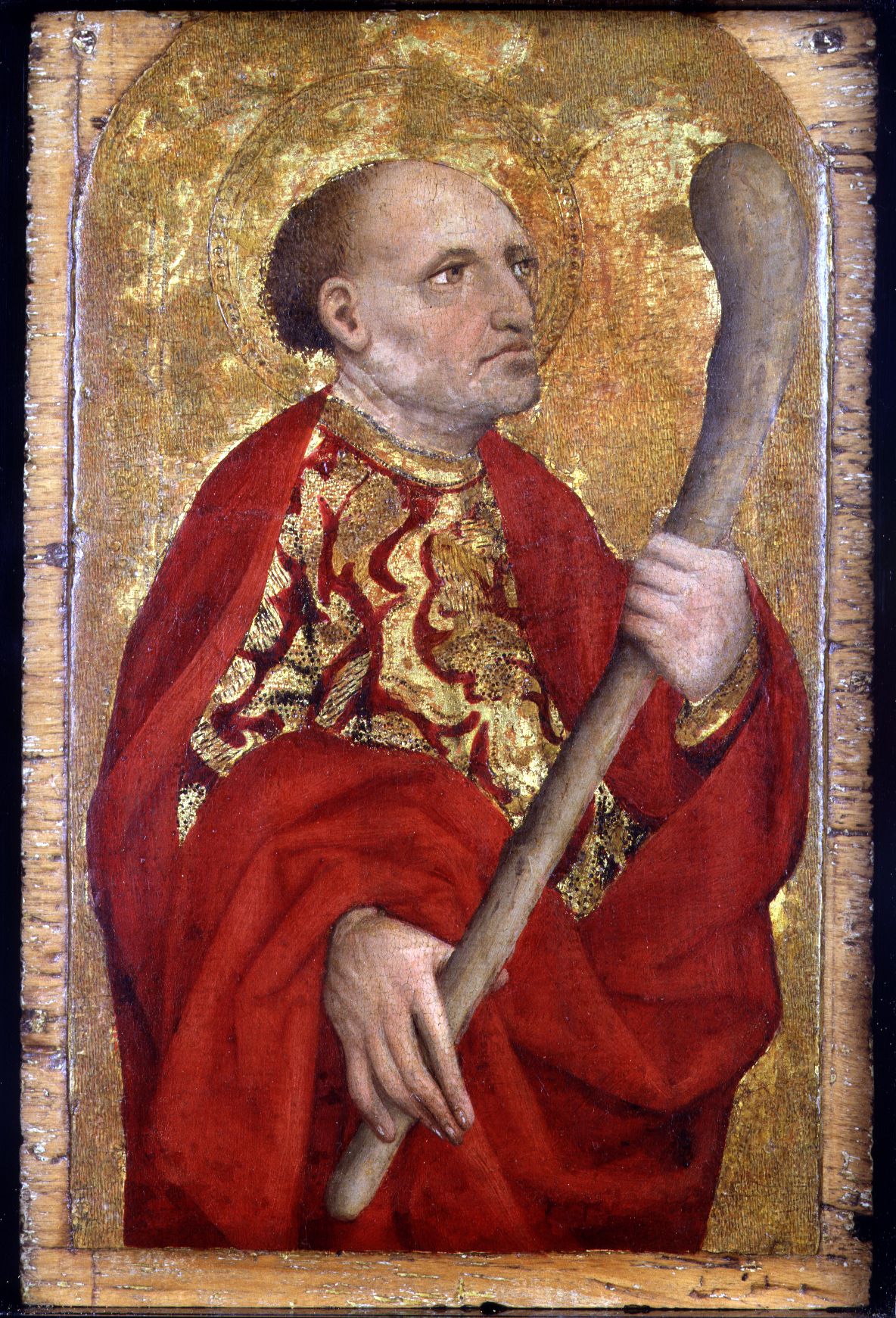

Supplement: Supplementary file 1 [file molecules-29-06043-s001.zip › Figure S6.jpg]

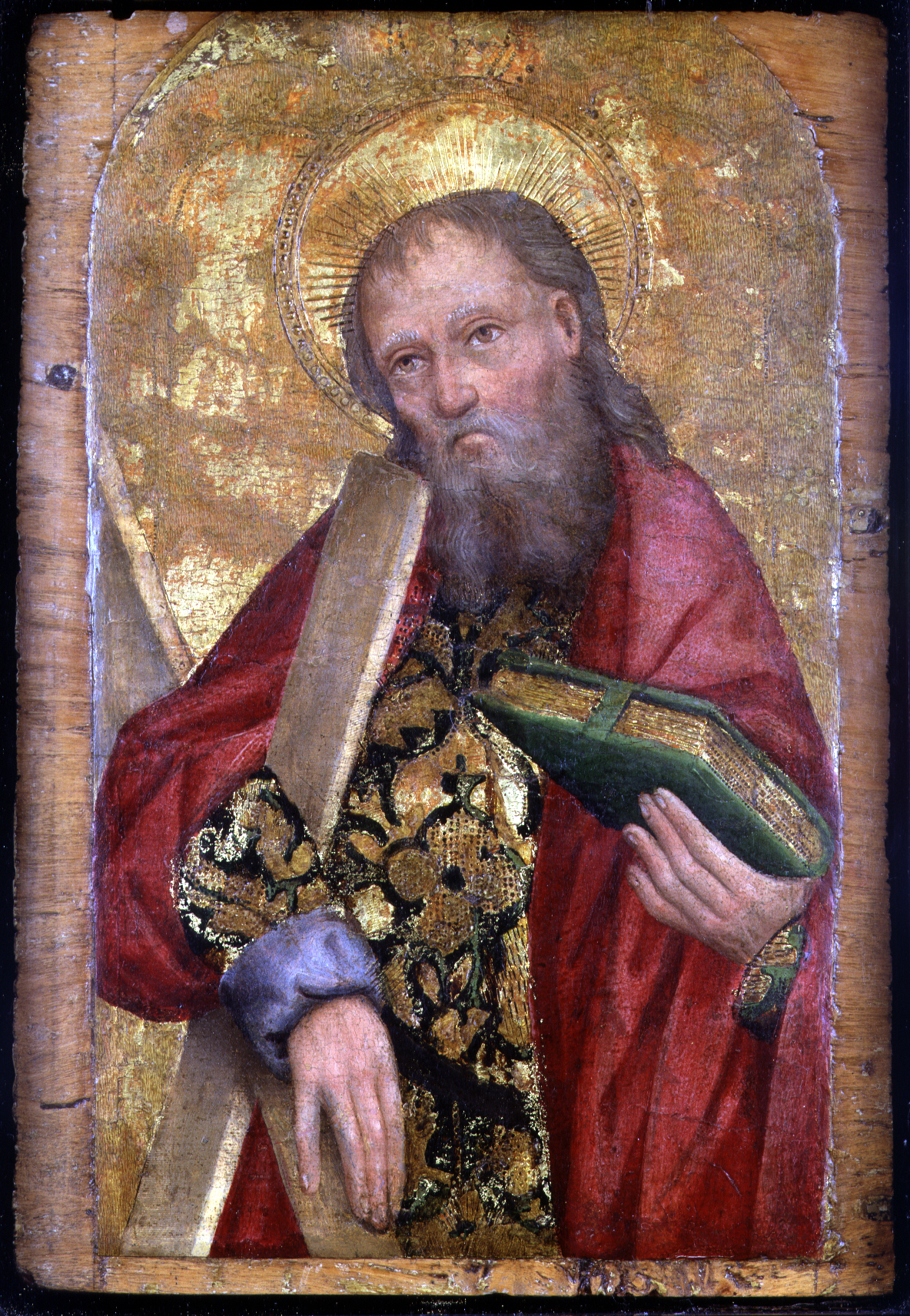

Supplement: Supplementary file 1 [file molecules-29-06043-s001.zip › Figure S7.jpg]

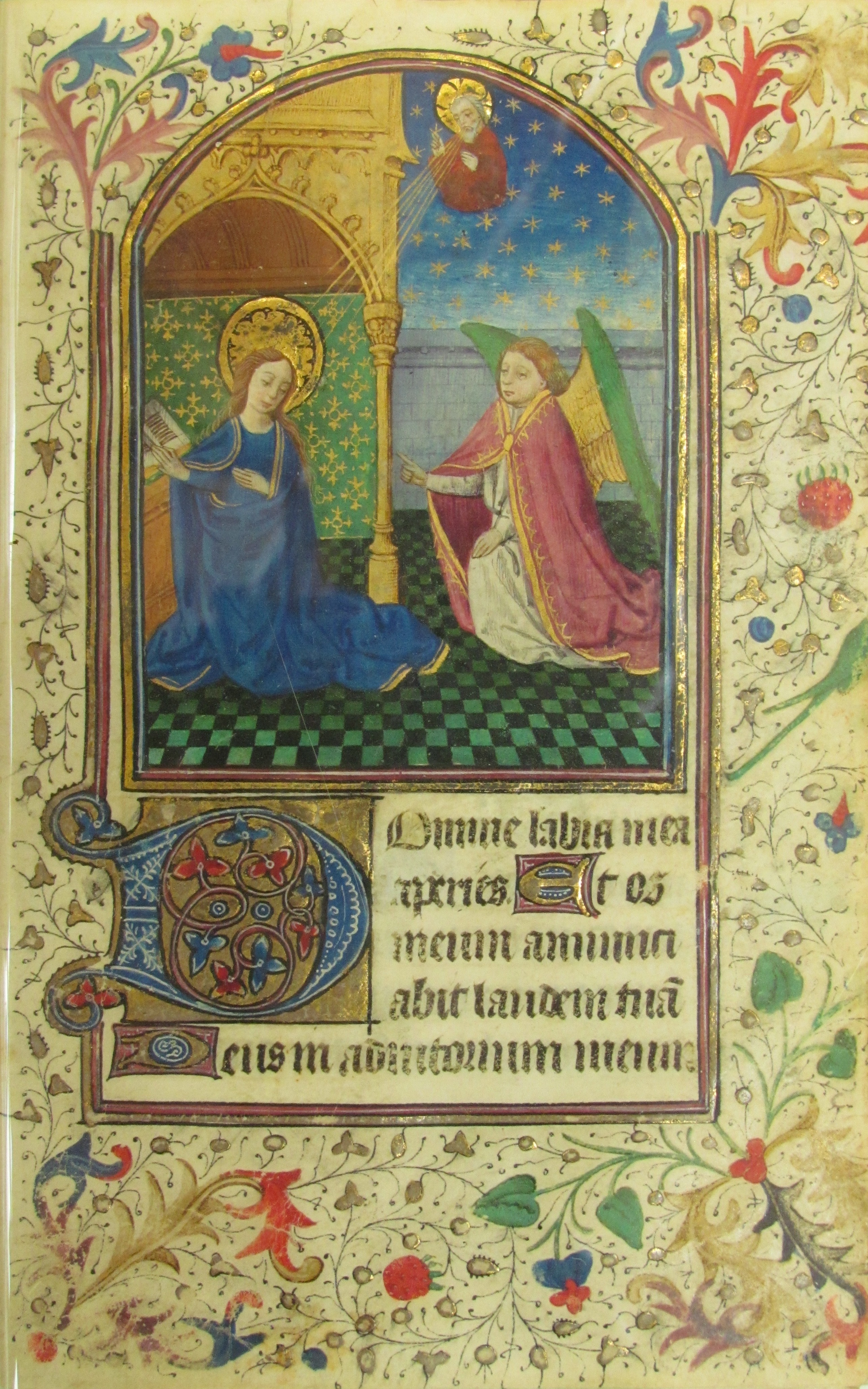

Supplement: Supplementary file 1 [file molecules-29-06043-s001.zip › Figure S8.JPG]

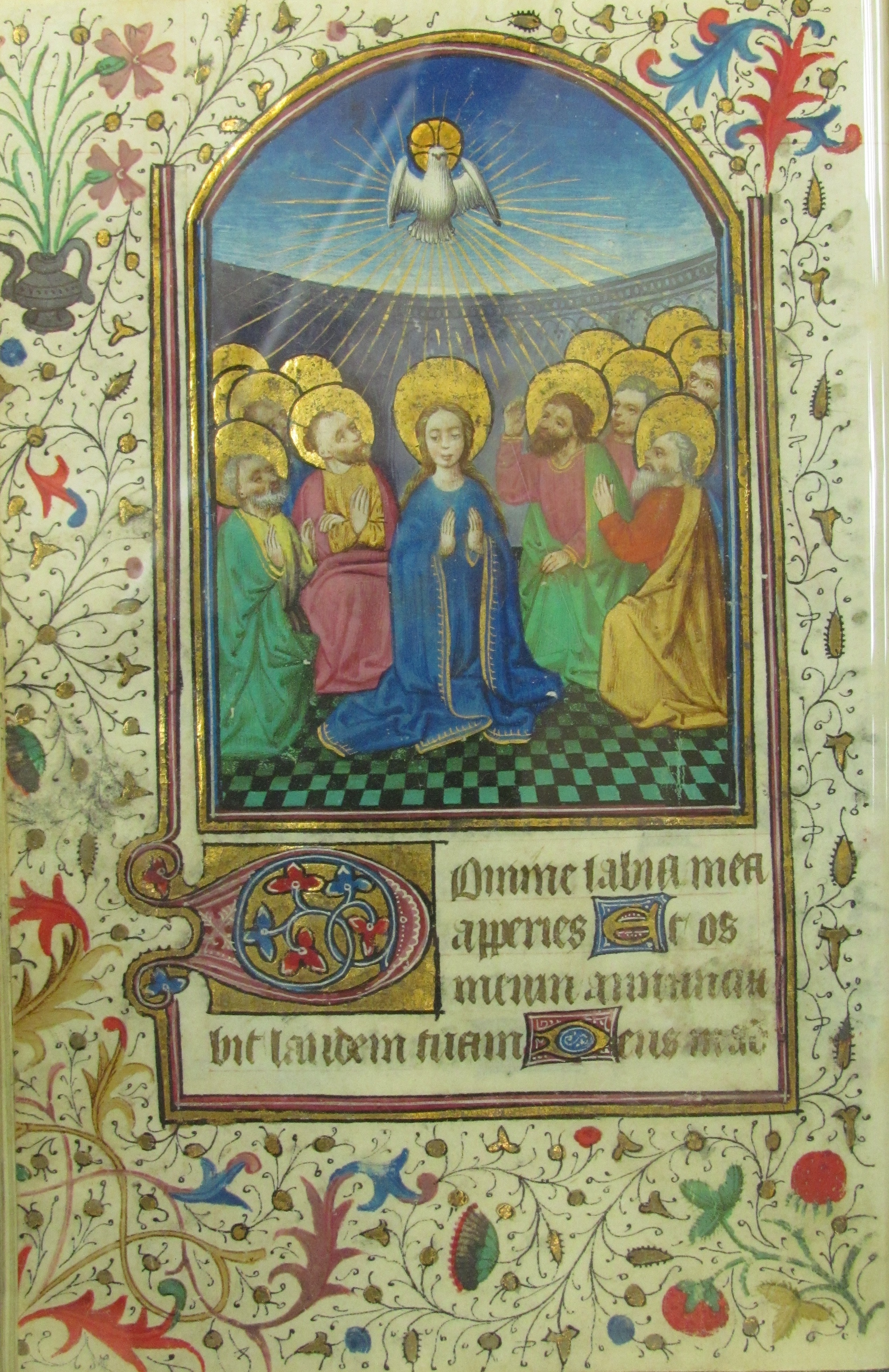

Supplement: Supplementary file 1 [file molecules-29-06043-s001.zip › Figure S9.JPG]
